# Cardiomyocyte-specific knockout of ADAM17 ameliorates left ventricular remodeling and function in diabetic cardiomyopathy of mice

**DOI:** 10.1038/s41392-022-01054-3

**Published:** 2022-08-01

**Authors:** Fei Xue, Jing Cheng, Yanping Liu, Cheng Cheng, Meng Zhang, Wenhai Sui, Wenqiang Chen, Panpan Hao, Yun Zhang, Cheng Zhang

**Affiliations:** 1 The Key Laboratory of Cardiovascular Remodeling and Function Research, Chinese Ministry of Education, Chinese National Health Commission and Chinese Academy of Medical Sciences, The State and Shandong Province Joint Key Laboratory of Translational Cardiovascular Medicine, Department of Cardiology, Qilu Hospital, Cheeloo College of Medicine, Shandong University, Jinan, 250012 Shandong China; 2 Heart Center and Beijing Key Laboratory of Hypertension, Beijing Chaoyang Hospital, Capital Medical University, Beijing, 100020 China; 3 Cardiovascular Disease Research Center of Shandong First Medical University, Central Hospital Affiliated to Shandong First Medical University, Jinan, China

**Keywords:** Cardiovascular diseases, Cardiology

## Abstract

Angiotensin-converting enzyme 2 (ACE2) has proven beneficial in attenuating diabetic cardiomyopathy (DCM) but has been found to be a substrate of a disintegrin and metalloprotease protein-17 (ADAM17). However, whether ADAM17 plays a role in the pathogenesis and intervention of DCM is obscure. In this study, we created cardiomyocyte-specific knockout of ADAM17 (A17^α-MHCKO^) mice, and left ventricular dimension, function, pathology and molecular biology were assessed in ADAM17^fl/fl^ control, A17^α-MHCKO^ control, ADAM17^fl/fl^ diabetic and A17^α-MHCKO^ diabetic mice. Both differentiated H9c2 cells and neonatal rat cardiomyocytes (NRCMs) were used to explore the molecular mechanisms underlying the effect of ADAM17 on DCM. The results showed that protein expression and activity of ADAM17 were upregulated whereas the protein expression of ACE2 was downregulated in the myocardium of diabetic mice. Cardiomyocyte-specific knockout of ADAM17 mitigated cardiac fibrosis and cardiomyocyte apoptosis and ameliorated cardiac dysfunction in mice with DCM. Bioinformatic analyses detected a number of genes enriched in metabolic pathways, in particular the AMPK signaling pathway, expressed differentially between the hearts of A17^α-MHCKO^ and ADAM17^fl/fl^ diabetic mice. The mechanism may involve activated AMPK pathway, increased autophagosome formation and improved autophagic flux, which reduced the apoptotic response in cardiomyocytes. In addition, hypoxia-inducible factor-1α (HIF-1α) might act as an upstream mediator of upregulated ADAM17 and ADAM17 might affect AMPK signaling via α1 A-adrenergic receptor (ADRA1A). These results indicated that ADAM17 activity and ACE2 shedding were enhanced in DCM, which was reversed by cardiomyocyte-specific ADAM17 knockout. Thus, inhibition of ADAM17 may provide a promising approach to the treatment of DCM.

## Abstract (web summary)

Proposed mechanisms underlying the salutary effects of ADAM17 deficiency on diabetic cardiomyopathy. ADAM17 deficiency increases autophagosome formation and improves autophagic flux via reducing ACE2 shedding, activating AMPK pathway, and promoting TFEB nuclear translocation, which reduces the apoptotic response in cardiomyocytes and attenuates left ventricular remodeling and dysfunction in DCM of mice.

## Graphical abstract

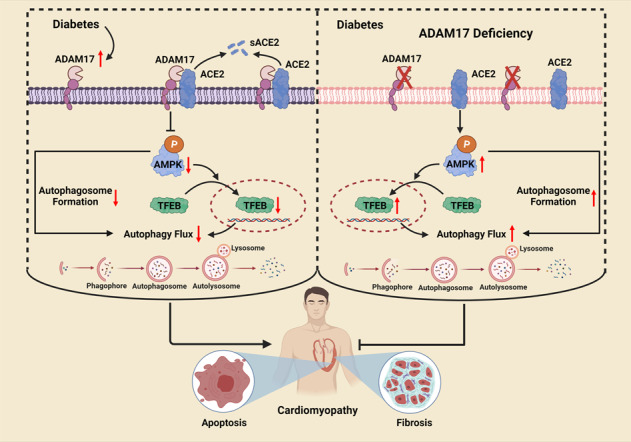

## Introduction

The prevalence of diabetes mellitus (DM) is on a rapid surge worldwide, and cardiovascular complications secondary to DM have emerged as a major challenge in the field of cardiovascular medicine^1^. A subset of diabetic patients exhibited cardiac systolic and diastolic dysfunction without hypertension and coronary artery disease, labeled diabetic cardiomyopathy (DCM)^2^. However, the pathological and molecular mechanisms of DCM are yet poorly understood, hindering the development of an effective therapeutic target.

The renin-angiotensin system (RAS) plays a key role in the pathogenesis of heart failure induced by many etiologies such as DCM, and angiotensin-converting enzyme inhibitors (ACEIs), angiotensin receptor blockers (ARBs) and aldosterone antagonists have proven to improve cardiac remodeling and dysfunction and become the cornerstone in the pharmacological treatment of heart failure^3,4^. However, these classical members of RAS as therapeutic targets have inherent limitations. Angiotensin II (Ang II) produced through chymase pathway cannot be inhibited by ACEIs, and intracellular Ang II cannot be blocked by ARBs^5,6^. In recent years, new members of RAS, such as angiotensin-converting enzyme 2 (ACE2), angiotensin-(1–7) [Ang-(1–7)], Mas receptor (MasR), angiotensin-(1–9) [Ang-(1–9)] and angiotensin IV (Ang IV), in the treatment of heart failure have received an increasing attention, and research work in our and other laboratories have discovered that ACE2-Ang-(1–7)-MasR, as a new axis of RAS, played a crucial role in the treatment of cardiovascular diseases^7^. ACE2 degrades Ang II to Ang-(1–7) and negatively regulates RAS activation in the heart^8^. Our group found that local ACE2 overexpression in the myocardium ameliorated left ventricular remodeling and dysfunction via an enhanced conversion of Ang II to Ang-(1–7) in a rat model of DCM^9^, and chronic infusion of Ang-(1–7) improved cardiac remodeling and function by reducing myocardial fibrosis, myocardial hypertrophy and cardiomyocyte apoptosis in a rat model of DCM^10^. These results suggested that activation of the ACE2-Ang-(1–7)-MasR axis may provide a promising new approach to the intervention of DCM.

A disintegrin and metalloprotease protein-17 (ADAM17), by shedding >70 substrates on the cell membrane, regulates multiple cellular responses^11^. Previous studies demonstrated that the serum level and activity of soluble ACE2 were increased in patients with heart failure, which correlated with cardiac dysfunction^12^. The mechanism may involve an increased activity of ADAM17 induced by RAS activation, which leads to enhanced shedding of ACE2, as an ADAM17 substrate, from cell membrane^13^. An early finding that the expression of ADAM17 was markedly increased in the myocardium of patients with dilated cardiomyopathy and hypertrophic obstructive cardiomyopathy inspired mechanistic studies in animal models^14,15^. However, results from experimental studies regarding the role of ADAM17 in cardiomyopathy were highly controversial. ADAM17 inhibition with small-interfering RNA prevented myocardial hypertrophy and fibrosis in Ang II-induced hypertensive mice as well as spontaneously hypertensive rats^16^. However, cardiomyocyte-specific ADAM17 knockdown was reported to aggravate cardiac hypertrophy by reducing integrin β1 cleavage in mice after transverse aortic constriction^17^. In mice with acute myocardial infarction, cardiomyocyte-specific knockdown of ADAM17 exacerbated left ventricular dilation and dysfunction by limiting the transcription of VEGFR2 as well as angiogenesis^18^. These contradictive results suggested that ADAM17 knockdown is a double-edged sword and plays a beneficial role only in conditions where RAS is highly activated. Hereto, there has been no study to report the therapeutic effects and underlying mechanisms of ADAM17 deficiency on DCM. As a wealth of evidence indicates that RAS is highly activated in DCM^19,20^, a series of in vivo and in vitro experiments were designed and performed to examine the effect of ADAM17 on the pathogenesis and treatment of DCM. This study provides a novel insight into the pathogenesis of and a promising therapeutic target for DCM. We created cardiomyocyte-specific knockout of ADAM17 (A17^α-MHCKO^) mice with or without diabetes and assessed left ventricular dimension, function, pathology and molecular biology in four groups of mice: ADAM17^fl/fl^ control, A17^α-MHCKO^ control, ADAM17^fl/fl^ diabetic and A17^α-MHCKO^ diabetic mice. We found that cardiomyocyte-specific knockout of ADAM17 mitigated cardiac fibrosis and cardiomyocyte apoptosis and ameliorated cardiac dysfunction in mice with DCM. We explored the mechanism underlying these beneficial effects, which may involve activated AMPK pathway, increased autophagosome formation and improved autophagic flux, which reduced the apoptotic response in cardiomyocytes. These results suggested that inhibition of ADAM17 may provide a promising approach to the treatment of DCM.

## Results

### Body weight, blood glucose and serum lipid profile

Body weight (BW), fasting blood glucose (FBG), total cholesterol (TC) and triglycerides (TG) were remarkably higher in A17^fl/fl^ DM group mice relative to those in the A17^fl/fl^ control group. By comparison, BW, FBG, and serum lipid levels did not differ between the A17^α-MHCKO^ DM and littermate A17^fl/fl^ DM group (Supplementary Table 1).

### Myocardial ADAM17 expression and activity were increased in diabetic hearts

Compared with controls, diabetic hearts showed markedly upregulated ADAM17 mRNA and protein expressions (Supplementary Fig. S2a–c) as well as an increased ADAM17 enzymatic activity (Supplementary Fig. S2d). Immunofluorescence analysis revealed more intensive staining of ADAM17 in the myocardium of diabetic mice than in control mice (Supplementary Fig. S2e, f). Besides, we used co-immunofluorescence staining to display ADAM17 expression in different cardiac cells and found that ADAM17 was abundantly expressed in cardiomyocytes, but sparsely expressed in fibroblasts and endothelial cells (Supplementary Fig. S2g).

### Cardiac remodeling and function were improved in A17^α-MHCKO^ diabetic mice

**Fig. 1:**
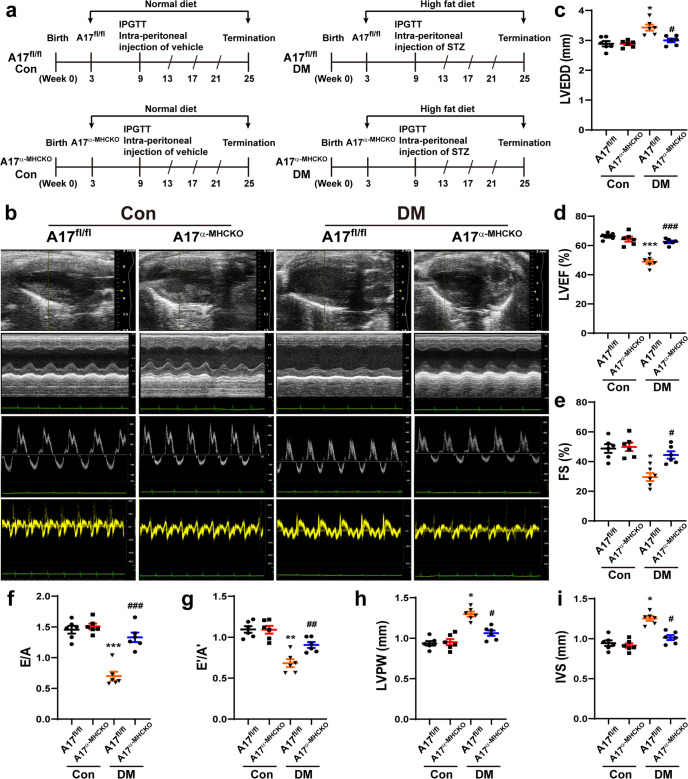
Experiment timeline and echocardiographic measurements in four groups of mice. **a** Experiment timeline in four groups of mice. **b** Representative echocardiographic images in mice: (1) Two-dimensional echocardiogram showing left ventricular long-axis view; (2) M-mode echocardiogram showing left ventricular dimensions; (3) Pulse-wave Doppler echocardiogram depicting diastolic mitral flow; (4) Tissue Doppler echocardiogram displaying mitral annular velocities. **c** Measurements of left ventricular end-diastolic diameter (LVEDD) in mice. **d** Measurements of left ventricular ejection fraction (LVEF) in mice. **e** Measurements of left ventricular fractional shortening (FS) in mice. **f** Measurements of the ratio of early to late diastolic mitral flow velocities (E/A) in mice. **g** Measurements of the ratio of early to late diastolic mitral annular velocities (E'/A') in mice. **h** Measurements of left ventricular posterior wall (LVPW) thickness in mice. **i** Measurements of interventricular septum (IVS) thickness in mice. Data were expressed as mean ± SEM. *P < 0.05, **P < 0.01, ***P < 0.001 vs. the A17^fl/fl^ control group; ^#^*P* < 0.05, ^##^*P* < 0.01, ^###^*P* < 0.001 vs. the A17^fl/fl^ DM group; *n* = 6.

Echocardiographic measurements of LVEDD, LVPW, and IVS were increased whereas LVEF, FS, E/A, and E’/A’ were decreased in the A17^fl/fl^ DM group compared with the A17^fl/fl^ control group (Fig. 1b–i). However, these echocardiographic values were all reversed in the A17^α-MHCKO^ DM group as compared with the A17^fl/fl^ DM group. Thus, cardiomyocyte-specific knockout of ADAM17 ameliorated left ventricular remodeling and rescued systolic and diastolic function in diabetic mice (Fig. 1b–i).

### Myocardial fibrosis and apoptosis were attenuated in A17^α-MHCKO^ diabetic mice

**Fig. 2:**
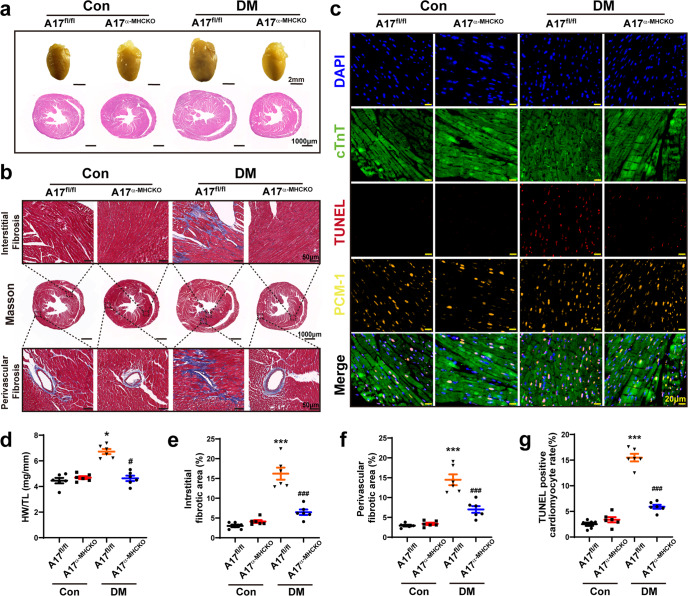
Histological and immunohistochemical staining in four groups of mice. **a** Representative images of hearts and myocardial cross-sections in four groups of mice: representative heart pictures (scale bar: 2 mm) were shown in the upper panel; Representative H&E staining of myocardial cross-sections (scale bar: 1000 μm) were shown in the lower panel. **b** Representative Masson’s trichrome staining of myocardial fibers. Masson’s trichrome staining of cardiac short-axis cross sectional areas (scale bar: 1000 μm) were shown in the middle panel. Dotted boxes in the middle panel indicated a local area in the low magnification field (scale bar: 1000 μm), which was enlarged in the high magnification field (scale bar: 50 μm) in the bottom panel. The interstitial fibrosis images were shown in the top panel and the perivascular fibrosis images were shown in the bottom panel. **c** Representative TUNEL-positive cardiomyocyte staining in mice (scale bar: 20 μm). **d** Quantitative analysis of heart weight/tibial length (HW/TL) ratio in mice, *n* = 6. **e** and **f** Quantification of the interstitial and perivascular fibrosis area in mice, *n* = 6. **g** Quantification of TUNEL-positive cardiomyocytes in mice, *n* = 6. Data were expressed as mean ± SEM. *P < 0.05, ***P < 0.001 vs. the A17^fl/fl^ control group; ^#^*P* < 0.05, ^###^*P* < 0.001 vs. the A17^fl/fl^ DM group.

Relative to the A17^fl/fl^ control group, the HW/TL ratio was upregulated in the A17^fl/fl^ DM group, whereas the HW/TL ratio in the A17^α-MHCKO^ DM group was downregulated relative to the A17^fl/fl^ DM group (Fig. 2a, d). In comparison with the A17^fl/fl^ control group, perivascular and interstitial fibrosis area in the myocardium displayed by Masson’s trichrome staining was substantially increased in the A17^fl/fl^ DM group, but significantly decreased in the A17^α-MHCKO^ DM mice group, relative to the littermate A17^fl/fl^ DM group (Fig. 2b, e, f). In order to assess the role of cardiomyocyte-specific knockout of ADAM17 in cardiomyocyte apoptosis, TUNEL staining together with staining of cardiomyocyte-specific marker cTnT and PCM-1 were performed. The proportion of TUNEL-positive apoptotic cardiomyocytes in the myocardium was markedly reduced in the A17^α-MHCKO^ DM group versus the littermate A17^fl/fl^ DM group mice (Fig. 2c, g).

**Fig. 3:**
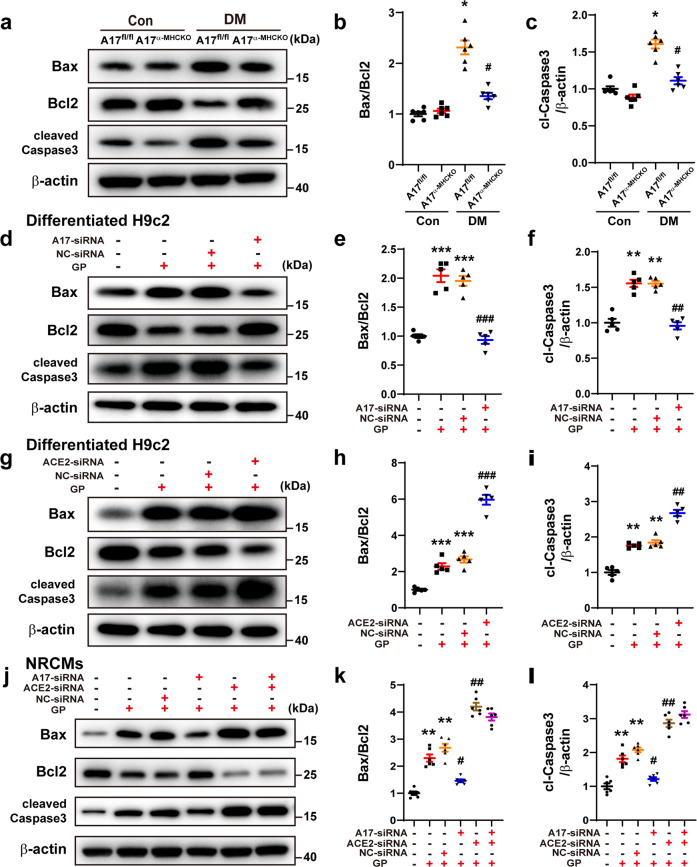
Effects of ADAM17 and ACE2 on apoptosis in mice, differentiated H9c2 cells and NRCMs. **a** Representative Western blot images of Bax, Bcl2, and cleaved caspase-3 expression in the myocardium of four groups of mice. **b**, **c** Quantitative analysis of Bax/Bcl2 and cleaved caspase-3 expression in four groups of mice, *n* = 6. Data were shown as mean ± SEM. *P < 0.05 vs. the A17^fl/fl^ control group; ^#^*P* < 0.05 vs^.^ the A17^fl/fl^ DM group. **d**–**f** Representative Western blot images of Bax, Bcl2, and cleaved caspase three expression and quantitative analysis of Bax/Bcl2 and cleaved caspase three expression in four groups of differentiated H9c2 cells treated with vehicle, GP, GP + NC-siRNA and GP + ADAM17-siRNA, respectively. Mean values were derived from five independent experiments. **g**–**i** Representative Western blot images of Bax, Bcl2, and cleaved caspase-3 expression and quantitative analysis of Bax/Bcl2 and cleaved caspase-3 in four groups of differentiated H9c2 cells treated with vehicle, GP, GP + NC-siRNA and GP + ACE2-siRNA, respectively. Mean values were derived from five independent experiments. Data were expressed as mean ± SEM. **P < 0.01, ***P < 0.001 vs. the vehicle group; ^##^*P* < 0.01, ^###^*P* < 0.001 vs. the GP + NC-siRNA group. **j** Representative Western blot images of Bax, Bcl2, and cleaved caspase-3 expression in six groups of NRCMs treated with vehicle, GP, GP + NC-siRNA, GP + ADAM17-siRNA, GP + ACE2-siRNA and GP + ADAM17-siRNA+ACE2-siRNA, respectively. **k**, **l** Quantitative analysis of Bax/Bcl2 and cleaved caspase-3 expression in six groups of NRCMs. Mean values were derived from six independent experiments. Data were presented as mean ± SEM. **P < 0.01 vs. the vehicle group; ^#^*P* < 0.05, ^##^*P* < 0.01 vs. the GP + NC-siRNA group.

To further evaluate the effect of cardiomyocyte-specific knockout of ADAM17 on apoptosis in the myocardium, the levels of Bax, Bcl2 and cleaved caspase-3 protein expression in the myocardium tissue were measured, which showed a significant downregulation in the A17^α-MHCKO^ DM group compared with A17^fl/fl^ DM group (Fig. 3a–c). Thus, cardiomyocyte-specific knockout of ADAM17 alleviated myocardial fibrosis and apoptosis in diabetic mice. In the in vitro experiment, the protein expression levels of Bax, Bcl2, and cleaved caspase-3 were detected in the differentiated H9c2 cells and neonatal rat cardiomyocytes (NRCMs), which were markedly downregulated in the ADAM17-siRNA group relative to the NC-siRNA group (Fig. 3d–f, Fig. 3j–l).

The undifferentiated and differentiated H9c2 cells showed two distinct features: First, H9c2 cells cultured in a medium containing 10% FBS presented a myoblast phenotype, with a mononucleated and spindle-to-stellate shape (Supplementary Fig. S3g). In contrast, H9c2 cells growing in the medium containing 1% FBS plus 1 μM RA for 7 days showed cell fusion and formation of more elongated multinucleated cells (Supplementary Fig. S3g), as described previously^21,22^. Second, differentiated H9c2 cells cultured in a 1% FBS media plus RA exhibited increased expression of cardiac-specific markers including cardiac troponin I and MYH6 (Supplementary Fig. S3h–k).

### ADAM17 was essential for cleaving ACE2 in vivo and in vitro

**Fig. 4:**
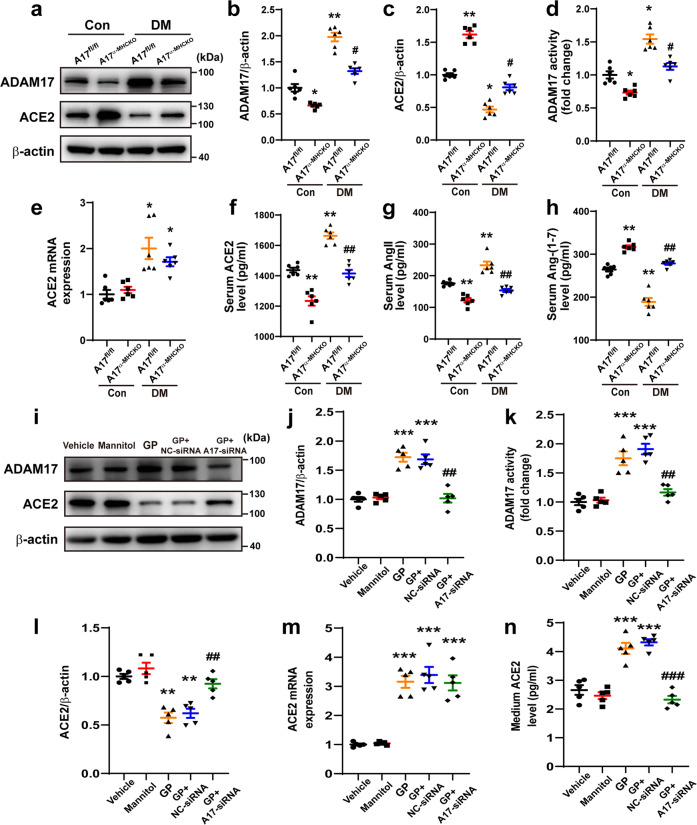
Serum levels of ACE2, Ang II, and Ang-(1–7) in mice and expression of ADAM17 and ACE2 in the myocardium of mice and in differentiated H9c2 cells. **a** Representative Western blot images of ADAM17 and ACE2 protein expression in the myocardium of four groups of mice. **b**, **c** Quantitative analyses of ADAM17 and ACE2 expression in the myocardium of mice, *n* = 6. **d** Quantitative analysis of ADAM17 activity in the myocardium of mice, *n* = 6. **e** Quantitative analysis of ACE2 mRNA expression in the myocardium of mice, *n* = 6. **f**–**h** Quantitative analysis of serum levels of ACE2, Ang II, and Ang-(1–7) in mice, *n* = 6. Data were shown as mean ± SEM. *P < 0.05, **P < 0.01 vs. the A17^fl/fl^ control group; ^#^*P* < 0.05, ^##^*P* < 0.01 vs. the A17^fl/fl^ DM group. **i**, **j** and **l** Representative Western blot images and quantitative analysis of ADAM17 and ACE2 protein expression in five groups of differentiated H9c2 cells treated with vehicle, mannitol, GP, GP + NC-siRNA and GP + ADAM17-siRNA, respectively. Mean values were derived from five independent experiments. **k** Quantitative analysis of ADAM17 activity in five groups of differentiated H9c2 cells treated as above. **m**, **n** Quantitative analysis of ACE2 mRNA level and ACE2 expression in the medium in five groups of differentiated H9c2 cells treated as above. Mean values were derived from five independent experiments. Data were shown as mean ± SEM. **P < 0.01, ***P < 0.001 vs. the vehicle group; ^##^*P* < 0.01, ^###^*P* < 0.001 vs. the GP + NC-siRNA group.

To explore the cleaving function of ADAM17, we examined mRNA levels of substrates of ADAM17 (TNF-α, TNF-αRI, TNF-αRII, IL-6, IL-6R, ALCAM, AREG, ERBB4, TGF-α, ICAM, VCAM, and ACE2) in the myocardium of diabetic and normal control mice (Supplementary Fig. S4a–l). ACE2 mRNA expression level was markedly upregulated in the myocardium of DCM mice, whereas the rest substrates did not differ in mRNA expression level between the two groups, suggesting that activated RAS is of paramount importance in the pathogenesis of DCM. The protein expression and activity of ADAM17 were downregulated in the myocardium of A17^α-MHCKO^ DM group as compared with the A17^fl/fl^ DM group (Fig. 4a, b, d), whereas the protein expression of ACE2 was upregulated in the myocardium of A17^α-MHCKO^ DM group compared with the A17^fl/fl^ DM group mice (Fig. 4a, b, c). Although ACE2 mRNA level was increased in DM mice than normal control, ADAM17 did not affect ACE2 mRNA expression in these mice (Fig. 4e), suggesting that ADAM17 affects only post-transcriptional expression level of ACE2.

ADAM17, as a negative regulator of RAS, mediates shedding of ACE2 from cell membrane and produces a soluble form of ACE2^13^. Thus, we detected the serum levels of ACE2, Ang II, and Ang-(1–7) in the in vivo experiment. In comparison with the A17^fl/fl^ control group, the soluble ACE2 level in the serum was markedly increased in A17^fl/fl^ DM group probably due to the increased expression and activity of ADAM17 in the myocardium. As a consequence of increased cleaving of ACE2, serum Ang II level was increased and serum Ang-(1–7) level was decreased in the A17^fl/fl^ DM group versus the A17^fl/fl^ control group (Fig. 4f–h). Conversely, the serum Ang II level was decreased while serum Ang-(1–7) level was increased in the A17^α-MHCKO^ DM group compared with the A17^fl/fl^ DM group (Fig. 4f–h). These results substantiated the key role of ADAM17 in regulating the ACE2-Ang II/Ang-(1–7) axis.

To further validate these in vivo results, differentiated H9c2 cells were treated with normal glucose, mannitol, GP, NC-siRNA, and ADAM17-siRNA, which showed that ADAM17 protein expression and activity were higher in the GP group than the mannitol group (Fig. 4i–k). In contrast, ACE2 protein expression was markedly lower in the GP group than the mannitol group, whereas ACE2 protein expression was markedly higher in the GP + ADAM17-siRNA group than the GP + NC-siRNA group (Fig. 4I, l). However, the mRNA level of ACE2 was increased with GP treatment, which was not affected by ADAM17-siRNA treatment (Fig. 4m), a finding in line with our in vivo results. The increased level of ACE2 in the culture medium after GP treatment as compared with the mannitol group was substantially suppressed with ADAM17-siRNA treatment (Fig. 4n), indicating that ADAM17 may cleave ACE2 into the medium.

To further assess whether ACE2 was connected with the effect of ADAM17 on apoptotic response, the related indicators of apoptosis were measured in differentiated H9c2 cells and NRCMs. The Bax/Bcl2 ratio and cleaved caspase-3 expression levels were increased by GP treatment, which was further elevated in the GP + ACE2-siRNA group (Fig. 3g–i). These results were opposite to that after ADAM17 knockdown (Fig. 3j–l). However, when gene knockdown of both ADAM17 and ACE2 were performed, the related indicators of apoptosis had no significant change as compared with ACE2-siRNA treatment alone (Fig. 3j–l). These results clearly indicated that cleaving ACE2 is an important function of ADAM17 and ACE2 plays a significant role in the effect of ADAM17 on apoptotic response.

### Metabolic pathway genes differentially expressed between A17^α-MHCKO^ and ADAM17^fl/fl^ diabetic mice

**Fig. 5:**
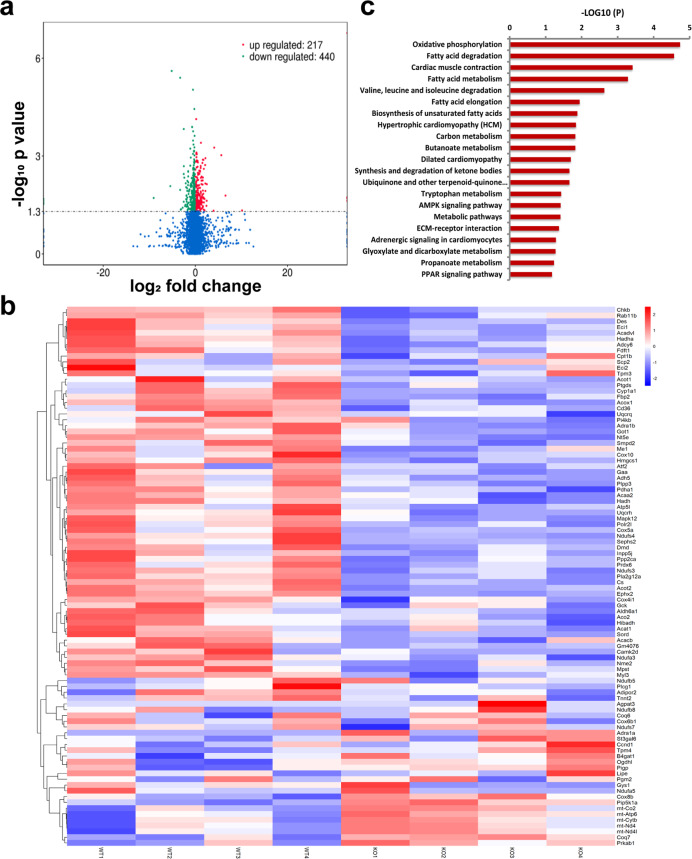
RNA-sequencing analysis of myocardial tissues in mice. **a** Volcano plot showing the transcript expression profiles in the hearts of A17^α-MHCKO^ and ADAM17^fl/fl^ diabetic mice, respectively. The horizontal line marked the threshold (*P* < 0.05) for defining upregulated (red dot) or downregulated (blue dot) genes in the myocardium of A17^α-MHCKO^ diabetic mice. The *x*-axis indicated log_2_ fold change and the *y*-axis indicated −log_10_ *P*-values. **b** Hierarchical clustering of the top 50 (ranked by *P*-values) genes differentially expressed in hearts from the A17^α-MHCKO^ and ADAM17^fl/fl^ diabetic mice. **c** KEGG pathway enrichment analysis of differential expression (DE) transcripts in the heart. The DE transcripts falling into the top pathways were summarized. The *x*-axis indicated –log10 (*P*-values) of the pathway and the *y*-axis displayed functional pathways

To obtain a comprehensive view of transcriptome changes associated with deficiency of ADAM17 in cardiomyocytes, we performed RNA sequencing from myocardial tissues isolated from the A17^fl/fl^ DM and A17^α-MHCKO^ DM mice. The results revealed that 217 genes were upregulated and 440 genes downregulated in the myocardium of the A17^α-MHCKO^ DM versus A17^fl/fl^ DM mice (Fig. 5a). The top 50 genes differentially expressed in the two groups of mice were depicted (Fig. 5b), which according to KEGG categories, involved oxidative phosphorylation, fatty acid degradation, cardiac muscle contraction, fatty acid metabolism, and AMPK signaling (Fig. 5c). In particular, we focused on the AMPK signaling pathway because of its central role in regulating glucose, lipid homeostasis and autophagy, and also because of a recent finding that AMPK served as a point of balance between physiological and pathological functions of the RAS^23^.

### ADAM17 regulated myocardial AMPK/mTOR pathway and TFEB expression in vivo and in vitro

**Fig. 6:**
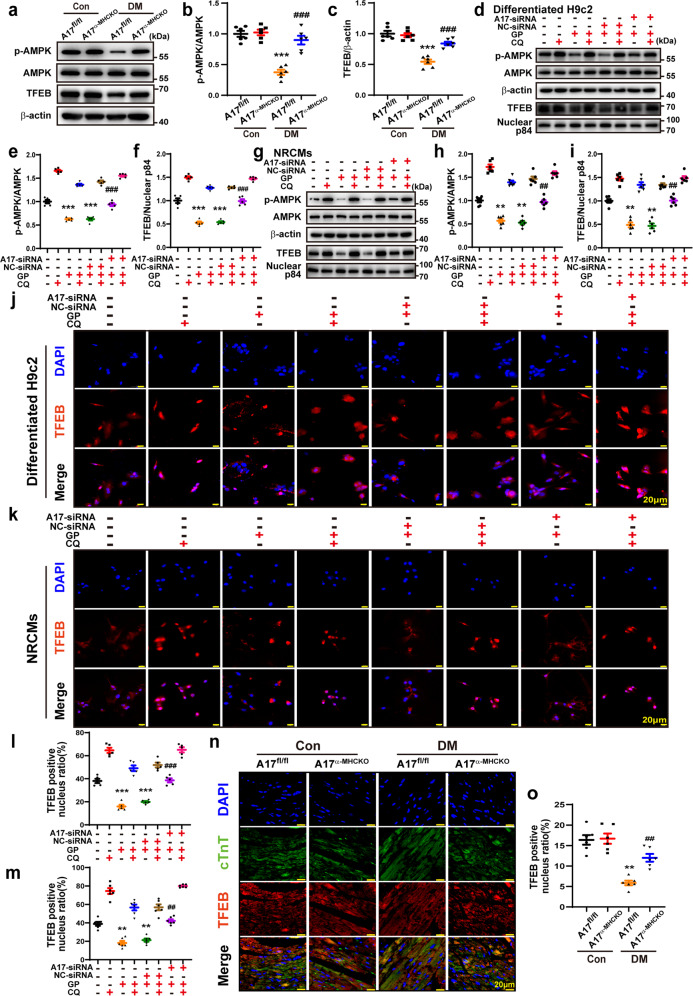
ADAM17 regulated the AMPK-TFEB pathway in vivo and in vitro. **a** Representative Western blot images of protein expression of phosphorylated AMPK, AMPK, and TFEB in the myocardium of four groups of mice. **b**, **c** Quantitative analysis of phosphorylated AMPK/AMPK and TFEB expression in the myocardium of four groups mice, *n* = 6. Data were expressed as mean ± SEM. ***P < 0.001 vs. the A17^fl/fl^ control group; ^###^*P* < 0.001 vs^.^ the A17^fl/fl^ DM group. **d**–**f** Representative Western blot images and quantitative analysis of phosphorylated AMPK, AMPK, and TFEB protein expression in eight groups of differentiated H9c2 cells treated with vehicle, GP, GP + NC-siRNA, and GP + ADAM17-siRNA, with and without additional chloroquine (CQ) treatment, respectively. Mean values were obtained from five independent experiments. **g**–**i** Representative Western blot images and quantitative analysis of phosphorylated AMPK, AMPK, and TFEB protein expression in eight groups of NRCMs treated as above. **j** Representative immunofluorescence staining of TFEB (red) in eight groups of differentiated H9c2 cells (scale bar: 20 μm) treated as above. **k** Representative immunofluorescence staining of TFEB (red) in eight groups of NRCMs treated as above (scale bar: 20 μm). **l** Quantification of TFEB-positive nucleus ratio in differentiated H9c2 cells. Mean values were derived from five independent experiments. **m** Quantification of TFEB-positive nucleus ratio in NRCMs. Six independent experiments were performed to derive the mean values. Data were expressed as mean ± SEM. **P < 0.01, ***P < 0.001 vs. the vehicle group; ^##^*P* < 0.01, ^###^*P* < 0.001 vs. the GP + NC-siRNA group. **n** Representative immunofluorescence staining of TFEB and cTnT in four groups of mice (scale bar: 20 μm). **o** Quantification of TFEB-positive nucleus ratio in cardiomyocytes of four groups of mice, *n* = 6. Data were presented as mean ± SEM. **P < 0.01 vs. the A17^fl/fl^ control group; ^##^*P* < 0.01 vs. the A17^fl/fl^ DM group

We found that ADAM17 deficiency reversed diabetes-induced inactivation of the AMPK pathway. The levels of AMPK phosphorylation and TFEB protein expression were remarkably decreased in the A17^fl/fl^ DM mice relative to the A17^fl/fl^ control mice whereas cardiomyocyte-specific knockout of ADAM17 significantly increased AMPK phosphorylation and TFEB protein levels in the A17^α-MHCKO^ DM mice (Fig. 6a–c). We future investigated the relationship between ADAM17 and AMPK phosphorylation after GP treatment in both differentiated H9c2 cells and NRCMs. The level of AMPK phosphorylation was dramatically decreased in the differentiated H9c2 cells of GP group relative to the control group, but returned to the level of the control group in the ADAM17 knockdown group (Fig. 6d–f). The results in NRCMs were consistent with those in differentiated H9c2 cells (Fig. 6g–i). Taken together, the results in vitro showed similar changes to those in vivo. Thus, ADAM17 played a critical role in the regulation of the AMPK signaling pathway in DCM. As TFEB drives the expression of autophagy and lysosomal genes^24^ and activation of AMPK may promote TFEB nuclear translocation^25^, we further examined whether ADAM17 affects lysosome function via TFEB nuclear translocation. The nuclear TFEB protein expression level was markedly decreased in the differentiated H9c2 cells of the GP group relative to the vehicle group (Fig. 6d, f), which was restored in the ADAM17-siRNA group relative to the NC-siRNA group. The results in NRCMs were consistent with those in differentiated H9c2 cells (Fig. 6g, i). In immunofluorescence analysis, TFEB nuclear translocation was significantly reduced in the GP group as compared with the vehicle group, but was virtually normalized in the ADAM17-siRNA group versus the NC-siRNA group (Fig. 6j–m). Thus, TFEB nuclear translocation was inhibited by GP treatment but enhanced by ADAM17 knockdown in both differentiated H9c2 cells and NRCMs. In addition, we examined TFEB nuclear translocation in myocardial tissues in four groups of mice, which showed similar results to those in the in vitro experiments (Fig. 6n, o).

**Fig. 7:**
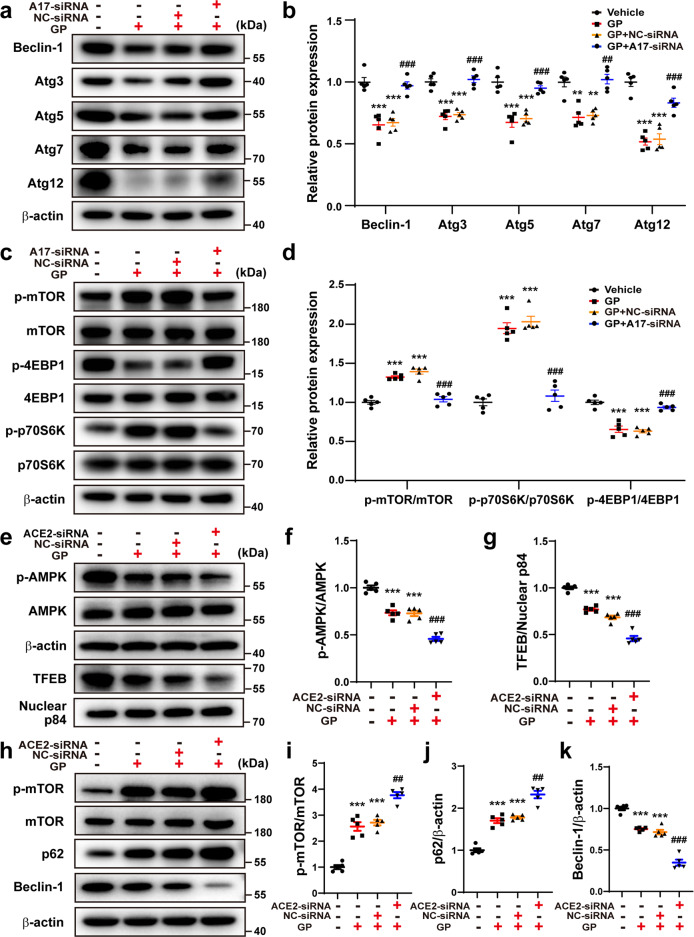
Effects of ADAM17 or ACE2 deficiency on autophagy-related proteins. **a**, **b** Representative Western blot images and quantitative analyses of protein expression of Beclin1, Atg3, Atg5, Atg7, and Atg12 in four groups of differentiated H9c2 cells treated with vehicle, GP, GP + NC-siRNA and GP + ADAM17-siRNA, respectively. **c**, **d** Representative Western blot images and analysis of phosphorylated mTOR, mTOR, phosphorylated p70S6K, p70S6K, phosphorylated 4EBP1 and 4EBP1 protein expression in differentiated H9c2 cells treated as above. Mean values were derived from 5 independent experiments. **e**–**g** Representative Western blot images and quantitative analysis of phosphorylated AMPK, AMPK, and TFEB protein expression in differentiated H9c2 cells treated with vehicle, GP, GP + NC-siRNA and GP + ACE2-siRNA, respectively. **h**–**k** Representative Western blot images and analysis of phosphorylated mTOR, mTOR, p62 and Beclin1 protein expression in differentiated H9c2 cells treated as above. Mean values were obtained from five independent experiments. Data were presented as mean ± SEM. *P < 0.05, **P < 0.01, ***P < 0.001 vs. the vehicle group; ^#^*P* < 0.05, ^##^*P* < 0.01, ^###^*P* < 0.001 vs. the GP + NC-siRNA group

The AMPK pathway regulates metabolic process, protein synthesis, cell proliferation, and autophagy via targeting the mammalian target of rapamycin (mTOR)^26^. Thus, to assess the effect of ADAM17 on the AMPK/mTOR pathway in cardiac autophagy, the mTOR pathway and its downstream activity, such as phosphorylated 4EBP1 (p-4EBP1) and phosphorylated p70S6K (p-p70S6K), were measured in differentiated H9c2 cells. Relative to the vehicle group, the protein expression of phosphorylated mTOR (p-mTOR) and p-p70S6K was significantly increased in the GP group, which was reversed in the ADAM17-siRNA group relative to the NC-siRNA group (Fig. 7c, d). Thus, the role of ADAM17 in the expression of AMPK and mTOR was opposite and consistent with previous data^26^. In addition, p-4EBP1 expression level was decreased in the GP group relative to the vehicle group, but was increased in the ADAM17-siRNA group versus the NC-siRNA group (Fig. 7c, d). It indicated that ADAM17 played a crucial role in the AMPK/mTOR signal pathway via regulating 4EBP1 and p70S6K protein phosphorylation.

### ADAM17 deficiency upregulated autophagy in vivo and in vitro

**Fig. 8:**
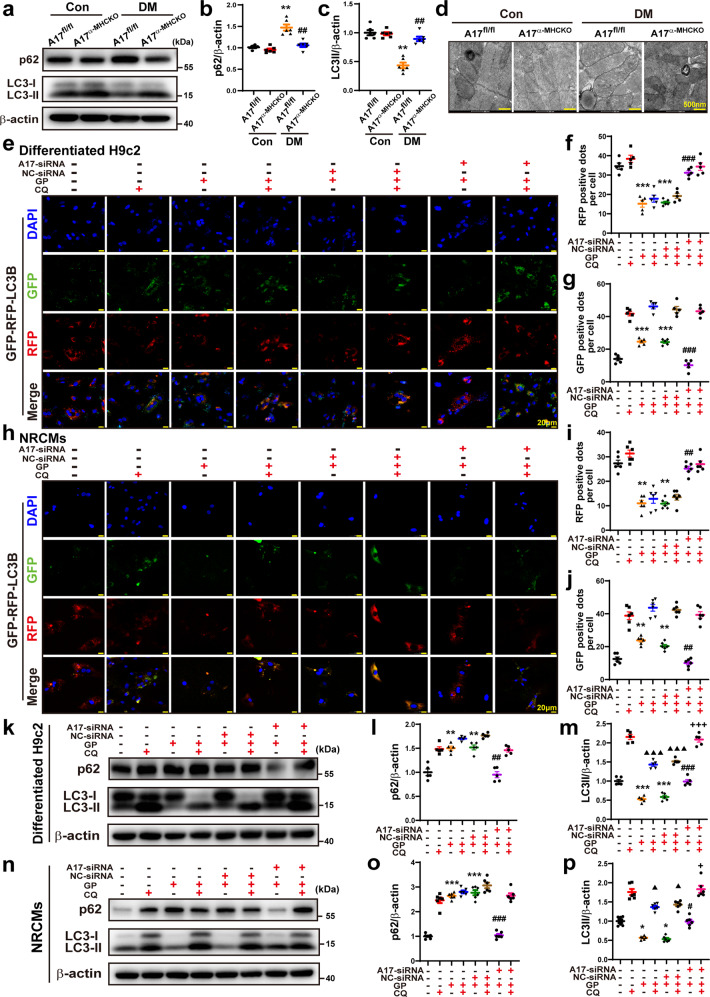
Effects of ADAM17 deficiency on cardiomyocyte autophagy in vivo and in vitro and on autophagic flux in differentiated H9c2 cells and NRCMs. **a** Representative Western blot images of autophagy-related protein expression, including p62 and LC3 in the myocardium of four groups of mice. **b**, **c** Quantitative analysis of protein expression of p62 and LC3II/β-actin in the myocardium of four groups of mice, *n* = 6. Data were shown as mean ± SEM. **P < 0.01, vs. the A17^fl/fl^ control group; ^##^*P* < 0.01 vs^.^ the A17^fl/fl^ DM group. **d** Representative transmission electron microscopy images of the myocardium in four groups of mice (scale bar: 500 nm). **e** Representative fluorescence images of green fluorescent protein (GFP; green), red fluorescent protein (RFP; red), GFP-RFP-LC3 (merged: yellow) and nuclei stained with DAPI (scale bar: 20 μm) in eight groups of differentiated H9c2 cells treated with vehicle, GP, GP + NC-siRNA and GP + ADAM17-siRNA, with and without additional CQ treatment, respectively. **f**, **g** Quantitative analysis of RFP-positive and GFP-positive dots per cell in differentiated H9c2 cells. Mean values were derived from five independent experiments. **h** Representative fluorescence images of GFP-RFP-LC3 in eight groups of NRCMs treated as above. **i**, **j** Quantitative analysis of RFP-positive and GFP-positive dots per cell in NRCMs. Mean values were obtained from six independent experiments. **k** Representative Western blot images of p62 and LC3 protein expression in eight groups of differentiated H9c2 cells treated as above. **l** and **m** Quantitative analysis of protein expression of p62 and LC3II/β-actin in differentiated H9c2 cells. Five independent experiments were performed to calculate the means. **n** Representative Western blot images of p62 and LC3 protein expression in eight groups of NRCMs treated as above. **o** and **p** Quantitative analysis of protein expression of p62 and LC3II/β-actin in NRCMs. Mean values were derived from six independent experiments. Data were expressed as mean ± SEM. *P < 0.05, **P < 0.01, ***P < 0.001 vs. the vehicle group; ^#^*P* < 0.05, ^##^*P* < 0.01, ^###^*P* < 0.001 vs. the GP + NC-siRNA group; ^▲^*P* < 0.05, ^▲▲▲^*P* < 0.001 vs. the CQ group; ^+^*P* < 0.05, ^+++^*P* < 0.001 vs. the GP + CQ + NC-siRNA group

AMPK-induced autophagy has been shown to attenuate the development of DCM^27^. In the process of lysosome degradation, p62 is degraded by proteolytic enzymes and an elevated level of p62 expression is generally considered to suggest the inhibition of autophagy activity^28^. We further examined whether autophagy was affected by ADAM17. Relative to the A17^fl/fl^ control group, the protein expression of p62 was increased in A17^fl/fl^ DM group, which was decreased in A17^α-MHCKO^ DM mice (Fig. 8a, b). In the in vitro experiment, p62 expression was downregulated in the ADAM17-siRNA group relative to the NC-siRNA group in both differentiated H9c2 cells and NRCMs (Fig. 8k–o). We also measured the ratio of LC3II/β-actin levels in vivo and in vitro. In the in vitro experiment, the LC3II/β-actin ratio was decreased in A17^fl/fl^ DM group as compared with the control group and returned to control levels in the A17^α-MHCKO^ DM group (Fig. 8a–c). In the in vitro experiment, the LC3II expression level was markedly upregulated in the ADAM17-siRNA group relative to the NC-siRNA group in both differentiated H9c2 cells and NRCMs (Fig. 8k–p). Transmission electron microscopy showed apparent swelling, vacuolation and myofibril disarray in the diabetic myocardium, which were ameliorated by cardiomyocyte-specific ADAM17 knockout (Fig. 8d).

### ADAM17 deficiency increased autophagic flux in cardiomyocytes

Analysis of autophagic flux rate is a key approach to measuring autophagy^29^. As a lysosomal acidification inhibitor to block the fusion of autophagosome and lysosome, CQ can cause autophagosome accumulation^30^. As ADAM17 played a crucial role in autophagy in cardiomyocytes, we further examined the role of ADAM17 in autophagosome turnover by treating differentiated H9c2 cells and NRCMs in the presence of either vehicle or CQ. Autophagic flux was imaged by fluorescence-tagged LC3B puncta in cells, where GFP-tagged LC3B indicates autophagic flux and RFP-tagged LC3B indicates autolysosomes^30^. As GFP is pH sensitive and quenched within the acidic environment of the lysosome, increased green fluorescence dots represent a blocked autophagic flux whereas increased red fluorescence dots reflect productive autophagosomes. In immunofluorescence analysis, RFP dots were markedly decreased in the GP group relative to the vehicle group, which was markedly reversed in the GP + ADAM17-siRNA group compared with the GP + NC-siRNA group (Fig. 8e, f). In contrast, GFP dots were increased in the GP group versus the vehicle group, which was reversed to the control level in the GP + ADAM17-siRNA group relative to the GP + NC-siRNA group (Fig. 8e, g). These results suggested that autophagic flux was inhibited by GP treatment and restored by ADAM17 knockdown.

As CQ blocks autophagic flux by inhibiting the fusion between autophagosome and lysosome, a late stage of autophagic flux, RFP dots showed no significant difference between two cell subgroups receiving vehicle and CQ treatment in each of the vehicle, GP, GP + NC-siRNA and GP + ADAM17-siRNA groups of differentiated H9c2 cells (Fig. 8e, f). In contrast, GFP dots were dramatically increased in the cell subgroup receiving CQ versus the subgroup receiving vehicle treatment in each of the four cell groups (Fig. 8g). Similar results were obtained in NRCMs (Fig. 8h–j). These results indicated that ADAM17 may inhibit autophagic flux at an early stage by attenuating the formation of autophagosome.

### Effect of ADAM17 or ACE2 deficiency on autophagosome formation-related proteins

In order to further assess whether ADAM17 affects the formation of autophagosome, the protein expression levels of Atg3, Atg5, Atg7, Atg12 and Beclin-1, which were associated with the process of autophagosome formation, were measured in differentiated H9c2 cells. Protein expression levels of Atg3, Atg5, Atg7, Atg12 were markedly downregulated in the GP group compared with the vehicle group, which was reversed in GP + ADAM17-siRNA group relative to the GP + NC-siRNA group (Fig. 7a, b). Beclin-1 protein expression showed similar changes in these cell groups (Fig. 7a, b).

To elucidate the effect of ACE2 on ADAM17-mediated autophagy, the autophagy-related proteins were measured in differentiated H9c2 cells treated with GP, GP + GP + NC-siRNA and GP + ACE2-siRNA. Relative to the GP + NC-siRNA group, protein expression level of p-AMPK, TFEB and beclin1 were decreased in the GP + ACE2-siRNA group. In contrast, the protein expression levels of p-mTOR and p62 were increased in the GP group relative to the vehicle group, which was further upregulated in the GP + ACE2-siRNA group versus the GP + NC-siRNA group (Fig. 7e–k). These results were opposite to those in cells with ADAM17 knockdown and indicated that ACE2 played an indispensable role in the effect of ADAM17 on cardiac autophagy.

### Effect of ADAM17 and ACE2 double-knockdown on cardiac apoptosis and autophagy

**Fig. 9:**
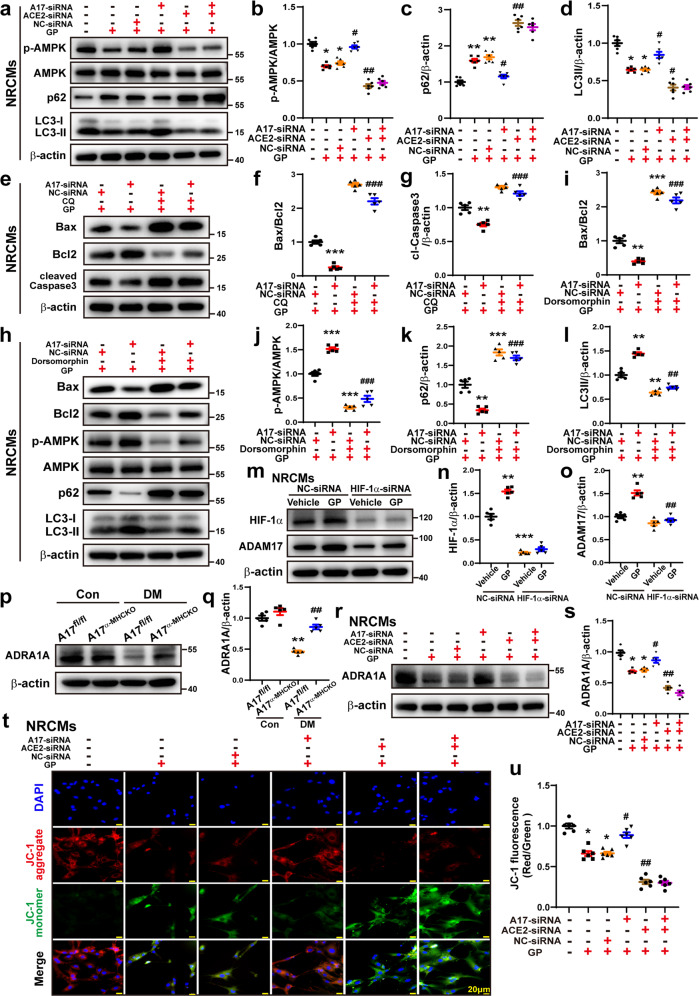
Effects of ADAM17 and ACE2 double knock-down and AMPK inhibitor on cardiac autophagy and apoptosis. **a** Representative Western blot images of protein expression of phosphorylated AMPK, AMPK, p62 and LC3 in six groups of NRCMs treated with vehicle, GP, GP + NC-siRNA, GP + ADAM17-siRNA, GP + ACE2-siRNA and GP + ADAM17-siRNA+ACE2-siRNA, respectively. **b**–**d** Quantitative analysis of p-AMPK/AMPK, p62 and LC3II/β-actin expression in six groups of NRCMs. Mean values were derived from six independent experiments. Data were expressed as mean ± SEM. *P < 0.05, **P < 0.01 vs. the vehicle group; ^#^*P* < 0.05, ^##^*P* < 0.01 vs^.^ the GP + NC-siRNA group. **e** Representative Western blot images of Bax, Bcl2, and cleaved Caspase-3 expression in four groups of NRCMs treated with GP + NC-siRNA+vehicle, GP + ADAM17-siRNA+vehicle, GP + NC-siRNA+CQ and GP + ADAM17-siRNA+CQ, respectively. **f**, **g** Quantitative analysis of Bax/Bcl2, and cleaved Caspase-3 expression in four groups of NRCMs. Five independent experiments were conducted to derive the means. Data were expressed as mean ± SEM. **P < 0.01, ***P < 0.001 vs. the GP + NC-siRNA+vehicle group; ^###^*P* < 0.001 vs. the GP + A17-siRNA group+vehicle group. **h** Representative Western blot images of Bax, Bcl2, p-AMPK, AMPK, p62 and LC3 expression in four groups of NRCMs treated with GP + NC-siRNA+vehicle, GP + ADAM17-siRNA+vehicle, GP + NC-siRNA+dorsomorphin, GP + ADAM17-siRNA+dorsomorphin, respectively. **i**–**l** Quantitative analysis of Bax/Bcl2, p-AMPK/AMPK, p62 and LC3II/β-actin expression in four groups of NRCMs. Mean values were derived from six independent experiments. Data were shown as mean ± SEM. **P < 0.01, ***P < 0.001 vs. the GP + NC-siRNA+vehicle group; ^##^*P* < 0.01, ^###^*P* < 0.001 vs. the GP + A17-siRNA group+vehicle group. **m** Representative Western blot images of HIF-1α and ADAM17 expression in four groups of NRCMs treated with vehicle+NC-siRNA, GP + NC-siRNA, vehicle+ HIF-1α-siRNA and GP + HIF-1α-siRNA, respectively. **n**, **o** Quantitative analysis of HIF-1α and ADAM17 expression in four groups of NRCMs. Five independent experiments were performed to calculate the means. Data were presented as mean ± SEM. **P < 0.01, ***P < 0.001 vs. the vehicle+NC-siRNA group; ^##^*P* < 0.01 vs. the GP + NC-siRNA group. **p**, **q** Representative Western blot images and analysis of ADRA1A protein expression in the myocardium of four groups of mice, *n* = 5. **P < 0.01 vs. the A17^fl/fl^ control group; ^##^*P* < 0.01 vs. the A17^fl/fl^ DM group. **r**, **s** Representative Western blot images and quantitative analysis of ADRA1A protein expression in NRCMs treated with vehicle^,^ G*P*, GP + NC-siRNA, GP + ADAM17-siRNA, GP + ACE2-siRNA and GP + ADAM17-siRNA+ACE2-siRNA, respectively. Five independent experiments were conducted to derive the means. Data were expressed as mean ± SEM. *P < 0.05 vs. the vehicle group; ^#^*P* < 0.05, ^##^*P* < 0.01 vs. the GP + NC-siRNA group. **t** and **u** Representative fluorescence images of JC-1 staining in NRCMs treated as above (scale bar: 20 μm). Quantitative analysis of JC-1 (JC-1 aggregates/JC-1 monomers) in NRCMs. Six independent experiments were performed to derive the mean values. Data were expressed as mean ± SEM. *P < 0.05, **P < 0.01 vs. the vehicle group; ^#^*P* < 0.05, ^##^*P* < 0.01 vs. the GP + NC-siRNA group

In order to further ascertain the effect of ADAM17 and ACE2 on cardiac apoptosis and autophagy, double-knockdown of ADAM17 and ACE2 was performed in NRCMs. The ratio of Bax/Bcl2 and protein expression of cl-caspase 3 showed no significant difference between GP + ACE2-siRNA and GP + ACE2 + A17-siRNA groups of NRCMs (Fig. 3j–l). Similarly, protein expression of p-AMPK, p62, and LC3II exhibited no difference between GP + ACE2-siRNA and GP + ACE2 + A17-siRNA groups of NRCMs (Fig. 9a–d). These results confirmed that ACE2 is a downstream mediator of ADAM17 and double knockdown of ADAM17 and ACE2 offered no extra effects over knockdown of ACE2 alone on cardiac apoptosis and autophagy.

To further clarify the effect of ADAM17 on apoptosis when the autophagic flux was inhibited, CQ was used to block the autophagic flux in the in vitro experiments in NRCMs. The ratio of Bax/Bcl2 and the protein expression level of cleaved caspase-3 were markedly decreased in GP + ADAM17-siRNA + vehicle group compared with the GP + NC-siRNA + vehicle group. However, when the autophagic flux was inhibited by CQ, the ratio of Bax/Bcl2 and the protein expression levels of cleaved caspase3 were markedly increased in GP + ADAM17-siRNA+CQ groups relative to GP + ADAM17-siRNA + vehicle group (Fig. 9e–g), indicating that ADAM17 knockdown in NRCMs reduced the apoptotic response via ameliorating the autophagic flux in cardiomyocytes.

### ADAM17 deficiency enhanced cell viability and mitochondrial membrane potential (MMP)

To evaluate the role of ADAM17 and ACE2 deficiency in cell viability, CCK8 assays were performed, which showed that the level of cell viability in the GP group was markedly reduced relative to the vehicle group, which was virtually reversed in the GP + ADAM17-siRNA group relative to the GP + NC-siRNA group. Notably, relative to the GP + NC-siRNA group, the cell viability level was further decreased in the GP + ACE2-siRNA group (Supplementary Fig. S5a, b).

JC-1 fluorescent probe is commonly used to detect MMP level, which is reflected by the ratio of red/green fluorescence. The MMP level was markedly lower in the GP group than the vehicle group, which was reversed by ADAM17 knockdown but resumed by ACE2 knockdown without additional effect of combined knockdown of ADAM17 and ACE2 (Fig. 9t–u).

### Effect of ADAM17 on ADRA1A/AMPK-mediated cardiac apoptosis and autophagy

In order to further clarify the role of AMPK in the impact of ADAM17 on apoptosis and autophagy, dorsomorphin, a selective AMPK inhibitor, was used in the in vitro experiments in NRCMs. The ratio of Bax/Bcl2 and the protein expression level of p62 were markedly upregulated whereas the expression levels of p-AMPK and LC3II were downregulated in GP + NC-siRNA + dorsomorphin group relative to the GP + NC-siRNA+vehicle group. These results were opposite to those in the GP + NC-siRNA+vehicle and GP + A17-siRNA+vehicle groups. However, the ratio of Bax/Bcl2 and the expression levels of p-AMPK, p62 and LC3II showed no significant difference between GP + NC-siRNA+dorsomorphin and the GP + A17-siRNA+dorsomorphin groups (Fig. 9h–l), indicating that ADAM17 knockdown in NRCMs suppresses cell autophagy and apoptosis via AMPK signaling pathway.

As shown by our results of RNA sequencing, there was a significant difference in the α1A-adrenergic receptor (ADRA1A) mRNA levels between myocardium samples of A17^α-MHCKO^ DM and the A17^fl/fl^ DM mice, and ADRA1A is involved in the AMPK signaling pathway according to KEGG categories (Fig. 5). As an upstream protein of AMPK, ADRA1A exerts a regulatory effect on the activity of AMPK^31^. To explore whether ADAM17 affected AMPK via ADRA1A, the protein expression levels of ADRA1A were measured in vivo and in vitro. ADRA1A protein expression was markedly lower in the A17^fl/fl^ DM group than in the A17^fl/fl^ control group, which was reversed in the A17^α-MHCKO^ DM relative to the A17^fl/fl^ DM group (Fig. 9p, q). The results in vitro showed similar changes in NRCMs (Fig. 9r, s).

Previous studies reported that high glucose upregulated ADAM17 through HIF-1α^32^, which was tested in the current study. The protein expression levels of both HIF-1α and ADAM17 levels were significantly increased in the GP + NC-siRNA group in comparison with the vehicle+NC-siRNA group in NRCMs. However, the protein expression levels of ADAM17 were dramatically decreased in the GP + HIF-1α-siRNA group versus the GP + NC-siRNA group (Fig. 9m–o), suggesting that the upregulated expression of ADAM17 in GP-treated cardiomyocytes was largely induced by HIF-1α.

## Discussion

In the present study, cardiomyocyte-specific ADAM17 knockout protected against left ventricular remodeling and dysfunction in DCM of mice. The underlying mechanisms involved attenuated ACE2 shedding and cardiomyocyte apoptosis, increased AMPK phosphorylation, TFEB nuclear translocation and autophagosome formation as well as a normalized autophagic flux. As far as we are aware of, our study is the first report of the therapeutic effects and underlying mechanisms of ADAM17 deficiency on DCM.

ADAM17 mediates ectodomain shedding of a set of substrates and the main substrate of ADAM17 varies with different disease models^33^. We examined mRNA levels of 12 substrates of ADAM17 including TNF-α, TNF-αRI, TNF-αRII, IL-6, IL-6R, ALCAM, AREG, ERBB4, TGF-α, ICAM, VCAM, and ACE2 in a mouse model of DCM and found that only ACE2 mRNA level showed a significant change in the DCM mice than the control mice, suggesting that ACE2 plays a central role in the pathogenesis of DCM. Our results lent support to the notion that ACE2 is the main substrate of ADAM17 in the setting of DCM. Although the ACE2 mRNA level was markedly upregulated in diabetic mice likely due to a compensatory mechanism, the ACE2 protein level in the myocardium of diabetic mice did not increase in comparison to that in control mice. This uncoupling phenomenon between ACE2 mRNA and protein expression levels is supportive of the post-translational regulation of ACE2 due to the proteolytic shedding by ADAM17, whereas the deficiency of ADAM17 resulted in preserved ACE2 on the cardiomyocyte surface. These findings were consistent with a previous report showing contradictory results between mRNA and protein expression levels of ACE2 in ADAM17^fl/fl^ mice receiving Ang II stimulation and suggesting a strong shedding effect of ADAM17 on ACE2^34^. To evaluate whether the apoptotic response induced by ADAM17 was associated with ACE2 in cardiomyocytes, we measured the expression levels of Bax, Bcl2, and cleaved caspase3 in ACE2-knockdown cardiomyocytes, and the results were opposite to that observed in ADAM17-knockdown cardiomyocytes. These findings demonstrated that ADAM17 was essential for cleaving ACE2, and ACE2 played a crucial role in the impact of ADAM17 on apoptotic response.

Previous studies reported that ACE2-knockout mice exhibited worsened cardiac function via inactivated myocardial AMPK^35^. AMPK is a key player in regulating a series of metabolic activities and AMPK activation has been confirmed to be beneficial for numerous metabolic diseases including DCM^36^. Recently, the research revealed that AMPK-mediated autophagic activation ameliorated oxidative stress, mitochondrial dysfunction, and cardiomyocyte apoptosis in diabetic mice^37^. The AMPK and mTOR pathways are interlinked with often opposite functions in sensing the availability of nutrients and energy and regulation of cell growth^38^, and the AMPK/mTOR signaling has been recognized as a key regulator of autophagy^26^. The correlation between the mTOR and AMPK pathways was evidenced by the discovery of eukaryotic initiation factor 4EBP1 and p70S6K, which are downstream targets of mTOR^26^. As the crucial regulators of mRNA translation and protein synthesis, both p70S6K and 4EBP1 participate in cell growth, cell survival, and apoptosis^39^. Thus, the phosphorylation level and activity of p70S6K and 4EBP1 are commonly used as an indicator of activated mTOR signaling pathway^26^. In our study, the role of ADAM17 knockdown in AMPK/mTOR pathway and its downstream p-4EBP1 and p-p70S6K were examined in differentiated H9c2 cells, which showed that cardiomyocyte-specific knockout of ADAM17 virtually reversed the inhibition of AMPK phosphorylation in DCM and ADAM17 played an important role in regulating AMPK/mTOR signal pathway.

As a new regulator of cell fate, the AMPK-TFEB pathway regulates the autophagic flux in the process of cell differentiation^40^. Upon stress, phosphorylated AMPK promotes TFEB transportation into the nucleus^41^, where TFEB binds promoters and controls numerous gene expression related to lysosomal functions such as phagocytosis, exocytosis, endocytosis, and autophagy^25^. A recent study showed that glucolipotoxicity diminished cardiomyocyte TFEB, which suppressed lysosomal autophagy and aggravated cardiac injury^30^. In a murine model of diabetes, cardiomyocyte autophagy was impaired^42^. Our results demonstrated that ADAM17 deficiency increased TFEB nuclear translocation both in vivo *and* in vitro, which reveals that ADAM17 might play a crucial role in the regulation of lysosome function.

As a scaffold protein containing multiple domains, P62 plays a critical role in regulating cell growth, cell survival, and inflammation^43^. In the process of lysosome degradation, p62 was degraded by proteolytic enzymes, and an elevated expression level of p62 has been deemed as an indicator of inhibited autophagy activity^28,43^. Beclin1 is a key regulator of autophagy and an inactive or dysfunctional Beclin1 leads suppressed autophagic process^44^. Thus, an abnormally low expression level of Beclin1 reflects a weakened autophagy^44^. Our results indicated that autophagic flux was suppressed in GP-treated cardiomyocytes and ADAM17 knockdown ameliorated autophagic flux.

Autophagy, as a cellular self-degradation process, maintains cellular integrity via eliminating damaged organelles and protein aggregates^45^. Current evidence indicates that activation of autophagy is a crucial cardioprotective mechanism that limits the accumulation of misfolded proteins^46^. As described previously, autophagy is a process including autophagy induction, cargo recognition, autophagosome formation, autophagosome-lysosome fusion, and substrate degradation^29^. One meaningful way to measure autophagy is to analyze the rate of autophagic flux rather than a snapshot look at autophagy at any one static point during the entire process^29,47^. CQ as a lysosomal acidification inhibitor to block fusion of autophagosome and lysosome, can cause autophagosome accumulation^30^. In this study, we administered CQ to clarify the specific process in autophagic flux ADAM17 may have impacts, and our results indicated that ADAM17 likely affected autophagosome formation in the early stage of autophagic flux.

LC3 which regarded as a marker of autophagosome in mammals, participates in the formation of autophagosome^48^ and the expression level of LC3 are proportional to the quantity of autophagic vacuoles^49^. Autophagy-related proteins participate in different steps of autophagy. Atg7, an E1-like enzyme, activates ubiquitin-like proteins Atg8 and Atg12 which undergo conjugation to form complexes that regulate the formation of autophagosome membranes^50^. It has been demonstrated that Atg5 and Atg7 were involved in the extension of autophagy membranes and formation of autophagic structures^51^, and Beclin-1, Atg3, Atg5, Atg7 and Atg12 participated in the process of autophagosome biogenesis^52^. In addition, the mRNA and protein expression of Atg5 and Atg7 were upregulated by inhibiting the mTOR pathway^46^. Our data demonstrated that expression of these autophagy-related proteins was downregulated in GP-treated cardiomyocytes, which was reversed by ADAM17 knockdown, suggesting that ADAM17 may play a significant role in the formation of autophagosomes.

Several questions regarding the pathological links in our animal experiments need to be addressed. First, why the expression and activity of ADAM17 were increased in diabetic mice? Previous studies showed that HIF-1α expression was increased in diabetic kidneys, which mediated the transcriptional upregulation of ADAM17^32^. Besides, the expression and activity of ADAM17 in macrophages were upregulated due to the activation of HIF-1α, leading to aggravated aortic dissection^53^. In our study, HIF-1α knockdown markedly attenuated the expression level of ADAM17, which was upregulated by GP treatment in NRCMs, suggesting that HIF-1α might act as an upstream mediator of upregulated ADAM17 in our diabetic mice. Second, how does ADAM17 affect AMPK phosphorylation? The results of RNA sequencing showed a significant difference in ADRA1A mRNA levels between A17^α-MHCKO^ DM and A17^fl/fl^ DM mice. It has been shown that ADRA1A directly regulated AMPK activation and thus, has been recognized as an upstream regulator of AMPK^31^. A previous study reported that activation of ADRA1A upregulated AMPK in skeletal muscle, resulting in an increased glucose uptake mediated by α1 adrenoceptor^54^. Our study revealed that ADRA1A protein level was markedly decreased in diabetic mice relative to control mice, which was reversed by cardiomyocyte-specific knockout of ADAM17. Similar results were derived from in vitro experiments in NRCMs. Thus, ADAM17 might affect AMPK signaling via ADRA1A. Third, how are autophagy and apoptosis logically related in diabetic mice? The casual relation between autophagy and apoptosis is highly controversial^55^ and depends on specific pathological background. Under some circumstances, such as in our mouse model of DCM, autophagy may constitute a stress adaptation against apoptosis and fibrosis, whereas in other conditions, such as acute myocardial infarction, autophagy may constitute an alternative cell-death pathway^55^. Autophagy, apoptosis and fibrosis can be caused by common diseases and signaling pathways, but in other instances, autophagy may affect apoptotic and fibrotic processes in a mutually exclusive manner^55^.

A major limitation of this study is a lack of preclinical studies using small molecule ADAM17 inhibitors in our mouse model or testifying our conclusions in human samples. Although some ADAM17 inhibitors have been screened for cancer research^56^, an ADAM17 inhibitor for attenuating DCM in mice has not been discovered. A major obstacle to a human sample study is due to the co-existence of extensive coronary atherosclerosis and DCM in almost all patients with advanced diabetes and it is difficult, if not impossible, to differentiate the effect of ADAM17 on ischemic and diabetic cardiomyopathy. Further validation studies are required in this area.

In conclusion, an increased ADAM17 expression and activity and decreased ACE2 expression were identified in the diabetic hearts. Cardiomyocyte-specific ADAM17-knockout attenuated left ventricular fibrosis and cardiomyocyte apoptosis and ameliorated cardiac remodeling and dysfunction in DCM of mice. The mechanism may involve activated AMPK pathway, increased autophagosome formation and improved autophagic flux, which reduced the apoptotic response in cardiomyocytes. These findings may provide a novel and promising target for the prevention and treatment of DCM.

## Supplementary information

Supplementary Materials-clean version

## Data Availability

All the datasets presented in the paper are available from the corresponding author upon reasonable request.
