# Supplementary material for: Cardiomyocyte-specific knockout of ADAM17 ameliorates left ventricular remodeling and function in diabetic cardiomyopathy of mice
Source: Signal Transduct Target Ther. 2022 Aug 1;7:259. doi: 10.1038/s41392-022-01054-3 (PMC9339545; doi:10.1038/s41392-022-01054-3)
Supplement: Supplementary file 1 — Supplementary Materials-clean version [file 41392_2022_1054_MOESM1_ESM.docx]

Supplementary Materials for

**Cardiomyocyte-specific knockout of ADAM17 ameliorates left ventricular remodeling and function in diabetic cardiomyopathy of mice**

Fei Xue ^a,1^, Jing Cheng ^a,c 1^, Yanping Liu ^a^, Cheng Cheng ^a^, Meng Zhang ^a,b^, Wenhai Sui ^a^, Wenqiang Chen ^a^, Panpan Hao ^a, *^, Yun Zhang ^a, b*^, Cheng Zhang ^a,b *^

Correspondence to: Cheng Zhang, email: zhangc@sdu.edu.cn, or Yun Zhang, email: zhangyun@sdu.edu.cn, or Panpan Hao, email: panda.how@sdu.edu.cn

**This PDF file includes:**

Materials and Methods

Supplementary Figures. S1 to S5

Supplementary Tables S1 to S4

Materials and Methods

**Ethics statement**

All experimental procedures were performed in accordance with the recommendations in the Guide for the Care and Use of Laboratory Animals published by the US National Institutes of Health (NIH publication No. 8023, revised 1978). All mice were housed in standard cages in a specific pathogen-free environment and kept on a 12-hr light/12-hr dark cycle with food and water freely available. All mice underwent euthanasia after experiments and hearts were extracted for further histological studies. All experiments were conducted under protocols approved by the Ethics Committee of Shandong University Qilu Hospital.

**Generation of cardiomyocyte-specific ADAM17-knockout mice**

ADAM17^flox/flox^ (ADAM17^fl/fl^) mice were purchased from the Jackson Laboratory and α-myosin heavy chain (α-MHC)-Cre mice were purchased from the Model Animal Research Center of Nanjing University. ADAM17^fl/fl^ mice were crossed with α-MHC-Cre mice to obtain A17^α-MHCKO^ mice through excising specifically exon 2 of the ADAM17 gene in cardiomyocytes. Cardiomyocyte-specific ADAM17-knockout mice (A17^α-MHCKO^) were generated at the predicted Mendelian ratio (Supplementary Figure S1a-S1b). Cardiomyocytes of mice were sorted by flow cytometry to verify ADAM17 knockout efficiency (Supplementary Figure S1c). A17^α-MHCKO^ mice exhibited approximately 80% reduction in the expression level of ADAM17 mRNA of sorted cardiomyocytes in comparison with littermate mice (Supplementary Figure S1d). The ADAM17 protein expression levels were significantly decreased in hearts of A17^α-MHCKO^ mice relative to ADAM17^fl/fl^ mice, whereas no significant difference was found in ADAM17 protein expression levels of liver and kidney between the A17^α-MHCKO^ and ADAM17^fl/fl^ mice (Supplementary Figure S1e-S1f).

**Animal model and grouping**

The flowchart of study design was presented in Figure 1a. Male ADAM17^fl/fl^ and A17^α-MHCKO^ mice were chosen by genotyping at the age of 3 weeks. The genotyping primers were listed in Supplementary Table 2. Male ADAM17^fl/fl^ and A17^α-MHCKO^ mice were randomly divided into four groups: ADAM17^fl/fl^ non-diabetic mouse group (A17^fl/fl^ control), A17^α-MHCKO^ non-diabetic mouse group (A17^α-MHCKO^ control), ADAM17^fl/fl^ diabetic mouse group (A17^fl/fl^ DM) and A17^α-MHCKO^ diabetic mouse group (A17^α-MHCKO^ DM). Mice with high-fat diet (HFD) had impaired glucose tolerance (Supplementary Figure S1). Mice in the control group were fed with normal chow diet and received an injection of vehicle (0.1 ml of citrate buffer, pH 4.5) at the age of 9 weeks. Mice in the A17^fl/fl^ DM group and A17^α-MHCKO^ DM group were fed with HFD and received an injection of streptozotocin (STZ) at the age of 9 weeks. The dose of STZ (Sigma, USA) was 75mg/kg, which was dissolved in 0.1 ml of citrate buffer (pH 4.5). Then mice were fed a normal chow in A17^fl/fl^ and A17^α-MHCKO^ control groups and HFD in A17^fl/fl^ DM and A17^α-MHCKO^ DM groups until the age of 25 weeks.

**Mouse model of diabetic cardiomyopathy**

Three-week-old male mice on C57 background were initially used to test the effects of high-fat diet (HFD) on glucose tolerance, who were randomly divided into two groups to receive a HFD (45% fat and 0.25% cholesterol) or a normal chow (NC) for 6 weeks. Then the intraperitoneal glucose tolerance test (IPGTT) and insulin tolerance test (ITT) were performed in all mice as described. At the age of 9 weeks, blood glucose levels of mice fed with HFD were significantly higher at 30 and 60 mins after glucose injection than those fed with NC diet (Supplementary Figure S1g). And the blood glucose level of mice fed with HFD was significantly higher at 30 min after insulin injection than those fed with NC diet (Supplementary Figure S1h). Thus, mice fed with HFD had impaired glucose tolerance and insulin sensitivity at 9 weeks of age. Thereafter, diabetes was induced in mice with HFD by intraperitoneal injection of streptozotocin (STZ; 75 mg/kg, Sigma, USA) dissolved in vehicle (0.1 ml of citrate buffer; pH 4.5). One week after STZ administration, blood glucose levels were measured after overnight fasting. Only mice with blood glucose levels ≥11.1 mM were included in the diabetic groups. The diabetic mice continued HFD feeding until age of 25 weeks.

**Intraperitoneal glucose tolerance test (IPGTT) and Intraperitoneal insulin tolerance test (IPITT) in cardiomyocyte-specific ADAM17-knockout mice**

In the last week of treatment, after fasting overnight (12 h), IPGTT and IPITT were performed in all mice. For IPGTT, mice were given glucose (2 mg/g body weight) by intraperitoneal injection, and blood glucose was measured before injection (0 min) and 15, 30, 60, and 120 mins after injection; For IPITT, mice were given insulin (1U/kg body weight) by intraperitoneal injection, and blood glucose was measured before injection (0 min) and 15, 30, 60, and 120 mins after injection. The results of both tests demonstrated that mice in the A17^fl/fl^ DM group and A17^α-MHCKO^ DM group had an impaired glucose tolerance and insulin sensitivity (Supplementary Figure S1i-S1j).

**Echocardiographic measurement**

After anesthesia with isoflurane in mice, transthoracic echocardiography was performed using Vevo2100 imaging system (VisualSonics, Toronto, Canada) by echocardiographers who were unaware of mouse grouping. Left ventricular end-diastolic diameter (LVEDD), left ventricular ejection fraction (LVEF), fractional shortening (FS), left ventricular posterior wall thickness (LVPW) and interventricular septum thickness (IVS) were measured by M-mode echocardiography in the left ventricular long-axis view. The early (E) and late (A) diastolic mitral flow velocities were measured by pulsed Doppler in the four-chamber view and the ratio of E/A was calculated. The early (E’) and late (A’) diastolic mitral annular velocities were measured by tissue Doppler imaging in the four-chamber view and the ratio of E’/A’ was derived.

**Histology and immunohistochemistry**

Heart weight (HW) and tibial length (TL) were measured in all mice to calculate the HW/TL ratio. Freshly excised hearts were fixed in 10% formalin, paraffin-embedded and sectioned (4μm thick). Masson’s trichrome staining was used to detect myocardial fibrosis and hematoxylin, and eosin (H&E) staining was performed to display cardiomyocyte morphology. ADAM17 protein localization in the myocardium was detected by immunofluorescence staining with an antibody against ADAM17. Primary antibodies against cardiac troponin T (cTnT), vimentin and CD31 were utilized to highlight cardiomyocytes, fibroblasts and endothelial cells, respectively.

**RNA-sequencing analysis**

RNA-sequencing analysis of myocardial samples was performed as previously described[^1^](#_ENREF_1). The samples were prepared and submitted to Novogene Bioinformatics Technology Co.Ltd (Beijing, China) for total RNA isolation, mRNA purification, library preparation, and sequencing. Paired-end sequence files (fastq) were mapped to the reference genome (GRCm38.102) using Hisat2 (Hierarchical Indexing for Spliced Alignment of Transcripts, version 2.0.5). The output SAM (sequencing alignment/map) files were converted to BAM (binary alignment/map) files and sorted using SAMtools (version 1.3.1). Gene abundance was expressed as fragments per kilobase of exon per million reads mapped (FPKM). Stringtie software was used to count the fragment within each gene, and TMM algorithm was used for normalization. Differential expression (DE) analysis of the transcripts between A17^α-MHCKO^ DM and ADAM17^fl/fl^ DM mice was performed using the DESeq package (Anders and Huber 2010) of R software (<http://www.rproject.org>). Differentially expressed mRNAs with p value <0.05 were considered as significantly modulated, and retained for further analysis. This choice is motivated by the decision to maximize the sensitivity of this analysis, in order to perform a massive screening and identify candidate genes to be validated with a wider sample population with real-time PCR analysis. Enrichment analysis was based on functional annotation of differentially expressed transcripts referring to the Kyoto Encyclopedia of Genes and Genomes (KEGG) pathway analysis.

**Co-localization of TUNEL staining and cardiomyocyte markers**

Myocardial sections (4μm thick) from four groups of mice were used for TUNEL staining. The paraffin sections required antigen retrieval with citrate buffer after deparaffinization and were then incubated with 3% H_2_O_2_ for 10 min at room temperature. Thereafter, the sections were blocked with 5% BSA for 30 min at 37°C and incubated with the corresponding primary antibodies against cTnT and pericentriolar material 1(PCM-1) at 4°C overnight, and then the tissue sections were incubated with appropriate fluorescent secondary antibodies for 30 min at 37°C after washing with PBS. Apoptosis of myocardial samples was detected by TUNEL using the In Situ Cell Death Detection Kit, TMR red (Roche, Germany) following the manufacturer's instructions. Cellular nuclei were stained with DAPI for 10 min at 37°C. The images were acquired with a fluorescence microscope (Ni-E, Nikon, Japan) with an excitation wavelength. The apoptosis ratio was expressed as the proportion of apoptotic cells to the total number of cells in the myocardium.

**Cell acquisition, culture and intervention**

Both H9c2 cells and neonatal rat cardiomyocytes (NRCMs) were used for *in vitro* experiments. H9c2 cells as a rat embryonic cardiomyocyte cell line has a potential to differentiate into adult cardiomyocytes under stimulation of a low level of serum content and all-trans retinoic acid (RA) in the cell culture media, as reported previously and thus applied in the present study [^2-4^](#_ENREF_2). NRCMs were isolated and cultured as described [^5^](#_ENREF_5).H9c2 cells (CRL-1446, RRID: CVCL_0286) were purchased from the American Type Culture Collection (Manassas, VA). The differentiation process of H9c2 myoblasts into cardiac phenotype was initiated by decreasing the percentage of serum in the media followed by RA (1μM, HY-14649, MCE) supplementation [^2^](#_ENREF_2). H9c2 cells were plated under non-differentiated conditions (10% FBS) or cell differentiated conditions (1% FBS + RA) in 6-well culture plates. The addition of RA to media containing 1% serum was performed daily in the dark for 7 days. The culture medium was replaced every 2 days. RA was prepared in DMSO and stored at -20°C in the dark to avoid degradation. Aliquots were used only once and diluted extemporaneously in the dark [^2-4^](#_ENREF_2). Cells were exposed to 5.5 mM glucose (normal glucose), 33.3 mM mannitol (hypertonic solution control group, 33.3/0.4-mM combination of glucose/palmitate (GP) [^6^](#_ENREF_6), scrambled negative control siRNA (NC-siRNA), ADAM17 siRNA, ACE2 siRNA or a combination of ADAM17 siRNA and ACE2 siRNA for 24 hours. siRNAs were transfected into cells by using Lipofectamine iMAX (Invitrogen, Carlsbad, CA). Palmitate was dissolved into 20% bovine serum albumin (BSA) to make a solution at a concentration of 0.4 mM. The palmitate solution was shaken well for 30 min in 55°C water bath and then filtered using 2 μm sterile filter. Only 20% BSA was used in the vehicle group.

**NRCM isolation and culture**

NRCMs were isolated from the neonatal rat heart. Briefly, the ventricular muscles of neonatal rats were isolated quickly, digested repeatedly with Collagenase Type 2 (LS004176, Worthinton) and HBSS, and the cells were harvested. Non-myocytes were removed by the differential adhesion method to obtain pure NRCMs. The NRCMs were resuspended with 85% DMEM (Gibco, USA), 8% fetal equine serum (909S051, Solarbio), 5% newborn calf serum (S9040, Solarbio), 100 U/ml penicillin and streptomycin (Gibco, USA), and 1% BrdU (19-160, Sigma) medium, and cultured in 95% O_2_ and 5% CO_2_ standard incubator at 37°C.

**Quantitative real-time RT-PCR**

Total RNA was extracted from freshly isolated murine heart samples and cells by using TRIzol Reagent (Invitrogen, Carlsbad, CA). Applied Biosystems cDNA Reverse Transcription and Takara SYBR RT-PCR kits were used for quantitative real-time RT-PCR. The primer sequences for ADAM17, ACE2 and β-actin genes were listed in Supplementary Table 3. Quantitative values were obtained by using the threshold cycle value (Ct) and relative mRNA expression levels were analyzed by the 2^-△△Ct^ method.

**Western blot analysis**

Proteins of murine hearts and cell extracts were separated by SDS-PAGE and transferred to polyvinylidene fluoride membranes for incubation with different primary antibodies (Supplementary Table 4) overnight at 4°C and appropriate secondary antibodies for 1 hour at room temperature. The levels of protein expression were normalized to that of β-actin.

**ADAM17 activity assay**

After treatment, proteins from murine hearts and cells were lysed in activity assay buffer (pH 7.4; 50 mM Tris HCl, 25 mM NaCl, 4% glycerol and 10 mM ZnCl_2_). ADAM17 activity was measured in duplicate for each sample by using the SensoLyte 520 TACE (α-Secretase) Activity Assay Kit (AnaSpec, Fremont, CA).

**Immunocytochemistry**

Cells were cultured in 12-well plates. To analyze TFEB localization, cells were incubated overnight at 4°C with TFEB antibody in a humidified chamber, washed with phosphate-buffered saline, then incubated with the secondary antibody for 30 min at 37°C. Nuclei were stained with DAPI. Images were acquired by laser scanning confocal microscopy (LSM710; Zeiss, Jena, Germany). Sections reacted with non-immune IgG, secondary antibody only, and no primary and secondary antibodies were used as negative controls.

**Autophagic flux assay**

Following exposure with different treatments, autophagic flux was assessed by treating cells with either vehicle or 20 μM chloroquine (CQ) for 3 hours in FBS deprived media. Cells were washed in PBS and then lysed by means of lysis buffer. The supernatant was collected and protein concentrations were determined using a BCA protein assay kit. Lysates were stored for subsequent biochemical analysis. Cells were transduced with GFP (green fluorescent protein)-RFP (red fluorescent protein)-LC3B Premo Autophagy Sensors (P36239; Thermo Fisher, USA) for 48 hours, and then exposed to different treatments. Thereafter, cells were treated with either vehicle or 20 μM CQ (HY-17589A, MCE, USA) for 3 hours to assess autophagic flux in FBS-deprived media. Cells were counterstained with DAPI and imaged at excitation/emission of approximately 504/511 nm for EGFP and 555/584 nm for TagRFP by using a fluorescence microscope.

**Cell viability assay**

Cell viability was assessed using the Cell Counting Kit-8 (CCK-8) (Abcam, ab228554, Cambridge, UK) according to the manufacturer’s instructions. The absorbance at 450 nm was measured in a microplate reader (BioTek, VT, USA).

**Mitochondrial membrane potential (MMP) assay**

JC-1 was assessed using the JC-1 mitochondrial membrane potential assay kit (M8650, Solarbio) following the manufacturer’s instructions. Briefly, NRCMs with a density of 1×10^6^ cells/mL were cultured in 12-well plates under 5% CO_2_ at 37°C. After different treatments, cells were stained with 1 mL JC-1 probe for 20 min in dark. The images were acquired with a fluorescence microscope (Ti-S, Nikon, Japan) and the fluorescence intensity of the JC-1 monomers and JC-1 aggregates was measured.

**Flow cytometry**

Myocardial tissues from mice were dissociated using the Neonatal Heart Dissociation Kit (No.130-098-373, Miltenyi, Germany) and the gentleMACS™ dissociator following the manufacturer's instructions. Cardiomyocytes were then fixed, permeabilized, and stained with anti-myosin heavy chain antibody (No.130-106-253, Miltenyi, Germany). Thereafter, cardiomyocytes were sorted with the BD Aria II (nozzie assy 100 um).

**Cell nuclear extraction**

Cell nuclei were separated by NE-PER Nuclear and Cytoplasmic Extraction Reagents (NO.78833, Thermo Fisher, USA) following the manufacturer's instructions for detecting nuclear protein TFEB. Phosphate-buffered saline (PBS, 0.1M phosphate, 0.15M sodium chloride, pH 7.2) was used as a buffer in the separation process.

**Statistical analysis**

The animal experimental groups of mice were labelled as A-D and thus data collection and analysis were carried out blindly. The data analyst was blinded to the treatment in each animal group and performed the statistical analysis independently, particularly with the histological procedures. Continuous data were expressed as mean ± standard error (SEM). After testing for normality and equality of variance, continuous data were compared by unpaired Student’s t-test (parametric analysis) between-group difference， and one-way analysis of variance (ANOVA) with Post hoc Bonferroni tests was used to compare data among multiple groups. After analysis of variance, post hoc tests were run only if F achieved the necessary level of statistical significance (*P* <0.05) and there was no significant variance inhomogeneity. The statistical significance of tests was determined by using SPSS 21.0 (SPSS Inc., Chicago, IL) and *P* <0.05 was considered statistically significant.

**References**

1 Xu, X. L., Cheng, T. Y., Yang, H., Yan, F. & Yang, Y. De novo sequencing, assembly and analysis of salivary gland transcriptome of Haemaphysalis flava and identification of sialoprotein genes. *Infect. Genet. Evol.* **32**, 135-142 (2015).

2 Ménard, C. *et al.* Modulation of L-type calcium channel expression during retinoic acid-induced differentiation of H9C2 cardiac cells. *J. Biol. Chem.* **274**, 29063-29070 (1999).

3 Branco, A. F. *et al.* Gene expression profiling of H9c2 myoblast differentiation towards a cardiac-like phenotype. *PloS one* **10**, e0129303 (2015).

4 Branco, A. F. *et al.* Isoproterenol cytotoxicity is dependent on the differentiation state of the cardiomyoblast H9c2 cell line. *Cardiovasc. Toxicol.* **11**, 191-203 (2011).

5 He, H. *et al.* Epigallocatechin-3-gallate pretreatment alleviates doxorubicin-induced ferroptosis and cardiotoxicity by upregulating AMPKα2 and activating adaptive autophagy. *Redox Biol*. **48**, 102185 (2021).

6 Trivedi, P. C. *et al.* Glucolipotoxicity diminishes cardiomyocyte TFEB and inhibits lysosomal autophagy during obesity and diabetes. *Biochim Biophys Acta.* **1861**, 1893-1910 (2016).

**Supplementary Figures**


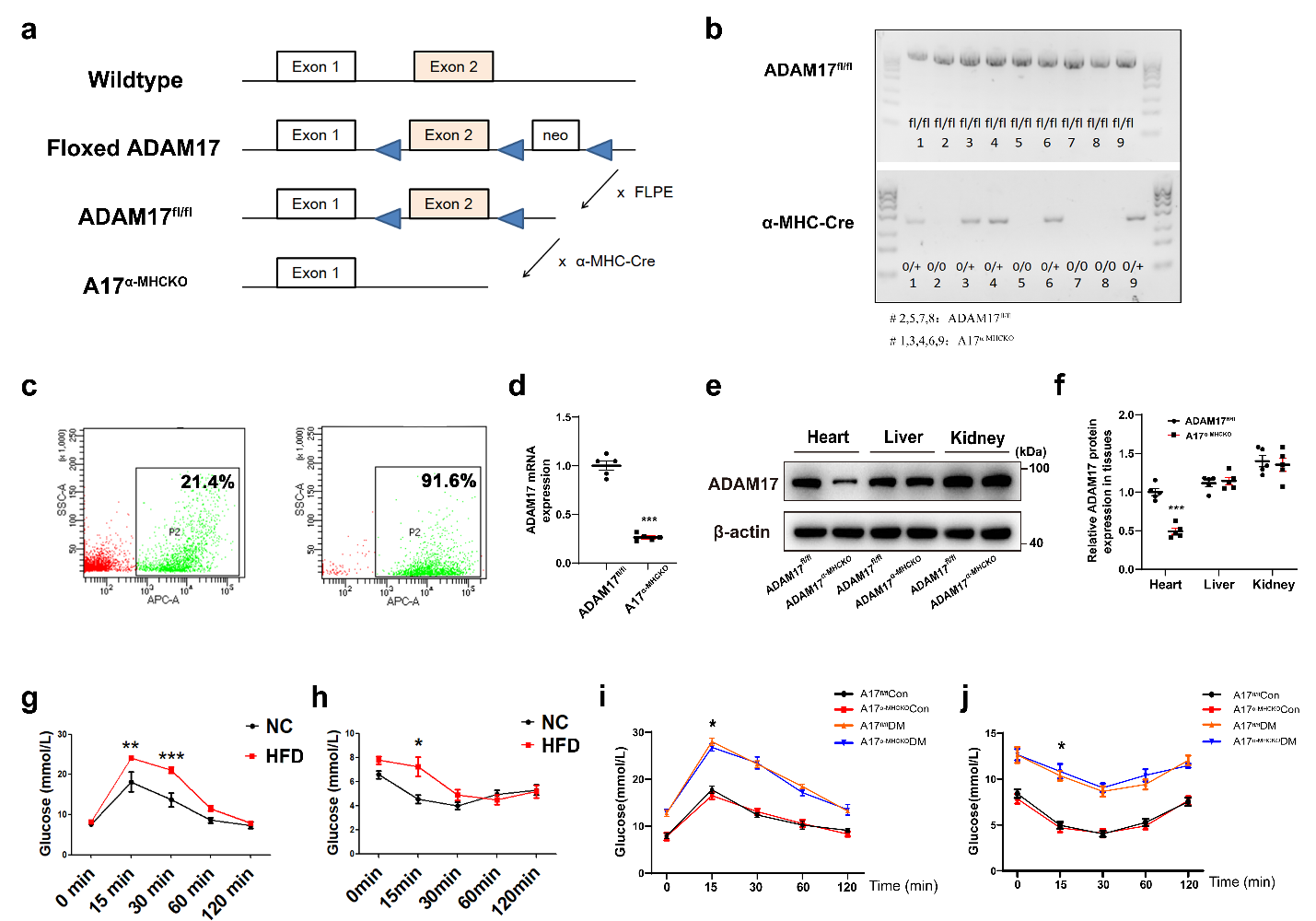


**Supplementary Figure S1.** **Genotyping of cardiomyocyte-specific ADAM17-knockout mice, and glucose and insulin tolerance tests in mice. (a)** Schematic diagram showing the breeding strategy. On introduction of cre-recombinase (α-MHC-cre), exon 2 of the ADAM17 gene was excised specifically in cardiomyocytes, allowing for generation of selective ADAM17 knockout mice (A17^α-MHCKO^). **(b)** Representative genotyping results for the A17^α-MHCKO^ and control littermates. Pups 1, 3, 4, 6, and 9 were identified as A17^α-MHCKO^ [ADAM17^fl/fl^ with cre recombinase (Cre+/0)], and pups 2, 5, 7, and 8 were control littermates [ADAM17^fl/fl^ without cre recombinase (Cre0/0)]. **(c)** Cardiomyocytes from adult mice were sorted by flow cytometry: (1) Proportion of myosin heavy chain positive staining cardiomyocytes in total dissociated cardiac cells; (2) Proportion of myosin heavy chain positive staining cardiomyocytes in sorted cardiomyocytes. **(d)** Quantitative analysis of ADAM17 mRNA expression in sorted cardiomyocytes of the A17^α-MHCKO^ and ADAM1^7fl/fl^ mice. Five independent experiments were performed to derive the mean values. **(e)** and **(f)** Representative Western blot images and quantitative analysis of ADAM17 protein expression in the heart, liver and kidney of the ADAM17^fl/fl^ and A17^α-MHCKO^ mice, n=5. Data were expressed as mean± SEM. ****P* <0.001 vs. the ADAM17^fl/fl^ mice. **(g)** Intraperitoneal glucose (2mg/g) tolerance test (IPGTT) in the ADAM17^fl/fl^ mice fed with either a high fat diet or normal diet, ***P* <0.01, ****P* <0.001 vs. normal diet at 15 and 30min, respectively. **(h)** Intraperitoneal insulin (1U/kg) tolerance test (IPITT) in the ADAM17^fl/fl^ mice fed with either a high fat diet or normal diet, **P* <0.05 vs. normal diet at 15min. **(i)** IPGTT (2mg/g) in four groups of mice in the last week of treatment, **P* <0.05 vs. the A17^fl/fl^ control group at 15 min. **(j)** IPITT (1U/kg) in four groups of mice in the last week of treatment, n=6. **P* <0.05 vs. the A17^fl/fl^ control group at 15 min. Data were expressed as mean± SEM. HFD: high fat diet; NC: normal diet.


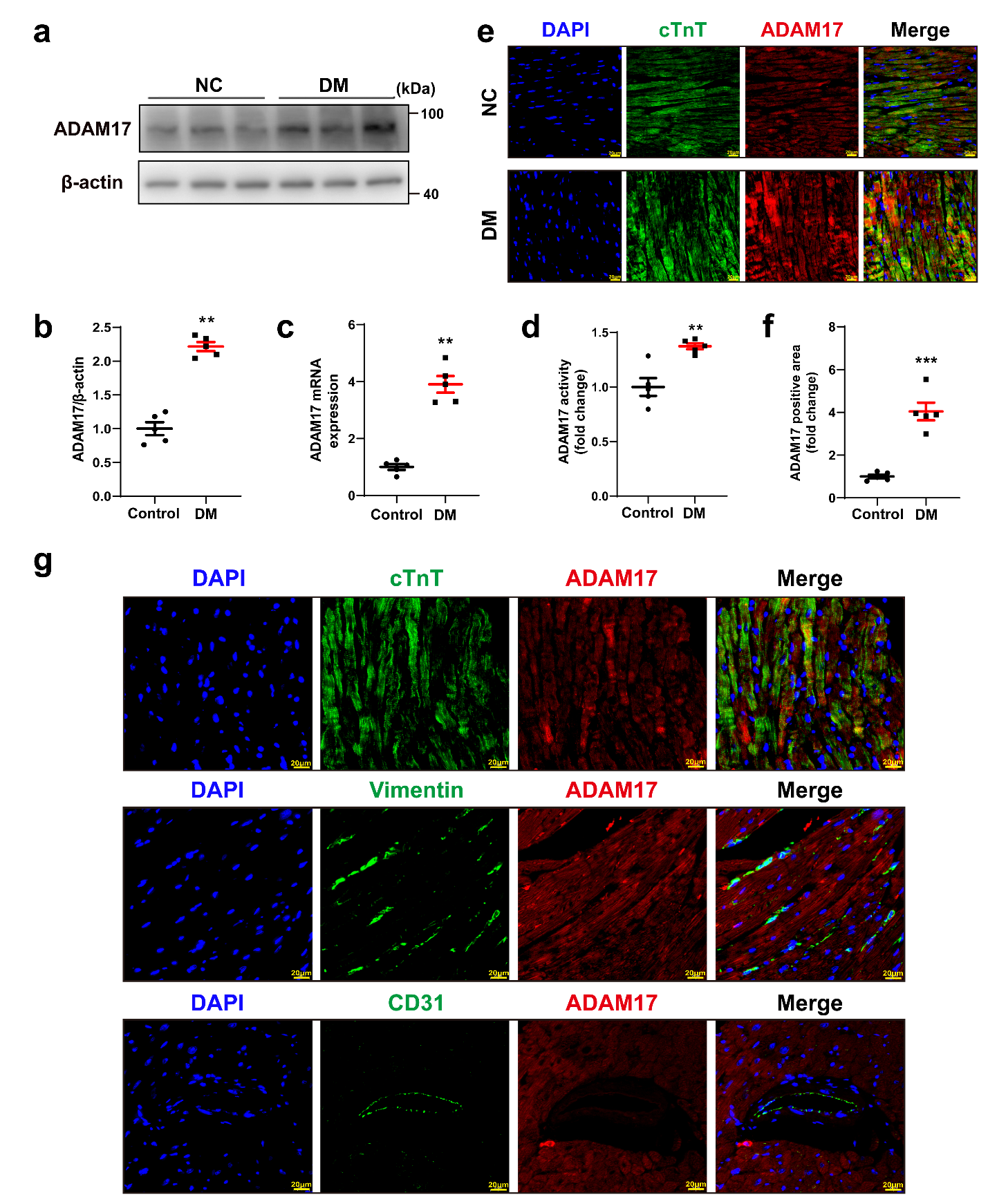


**Supplementary Figure S2.** **ADAM17 protein expression and activity in the myocardium of control and diabetic mice, and localization of ADAM17 in different cardiac cells of mice.** **(a)** Representative Western blot images of ADAM17 protein expression in the myocardium of control and diabetic mice. **(b)** Quantitative analysis of ADAM17 protein expression in the myocardium of control and diabetic mice. **(c)** Quantitative analysis of ADAM17 mRNA expression in the myocardium of control and diabetic mice. **(d)** Quantitative analysis of ADAM17 activity in the myocardium of control and diabetic mice. Data were expressed as mean± SEM. ***P* <0.01, ****P* <0.001 vs. the control group; n=5. **(e-f)** Representative immunofluorescence staining of ADAM17 protein expression in the myocardium of control and diabetic mice. Scale bar: 20μm. **(g)** Representative immunofluorescence staining of ADAM17 (red) in different cardiac cells (green). cTnT, Vimentin and CD31 were used as markers for cardiomyocytes, fibroblasts and endothelial cells, respectively (Scale bar: 20μm).


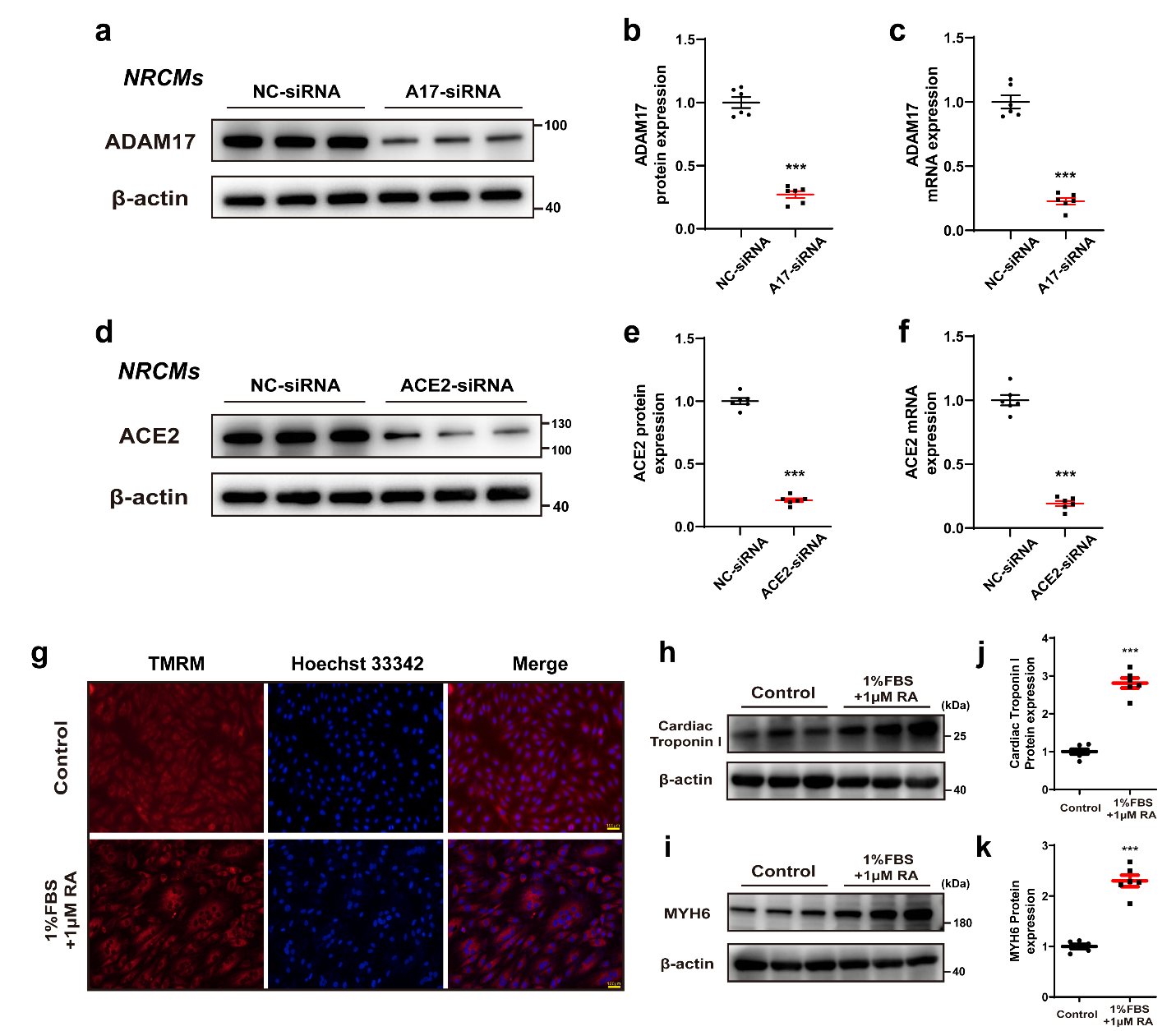


**Supplementary Figure S3.** **Efficacy of ADAM17 and ACE2 knockdown in NRCMs, and characterization of differentiated H9c2 cells**. **(a)-(c)** Representative Western blot images and quantitative analysis of ADAM17 protein and mRNA expression in two groups of NRCMs treated with NC-siRNA and ADAM17-siRNA. **(d)-(f)** Representative Western blot images and quantitative analysis of ACE2 protein and mRNA expression in two groups of NRCMs treated with NC-siRNA and ACE2-siRNA. Six independent experiments were performed to derive the mean values. Data were expressed as mean± SEM. ****P* <0.001 vs. the NC-siRNA group. **(g)** Representative images of differentiated H9c2 cells (scale bar: 100μm). **(h)** and **(i)** Representative Western blot images of the expression of two cardiac-specific markers, cardiac troponin I and MYH6, in differentiated and undifferentiated H9c2 cells. **(j) and (k)** Quantitative Western blot analysis of cardiac troponin I and MYH6 expression in differentiated and undifferentiated H9c2 cells. Six independent experiments were performed to derive the mean values. Data were expressed as mean± SEM. ****P* <0.001 vs. the control group.


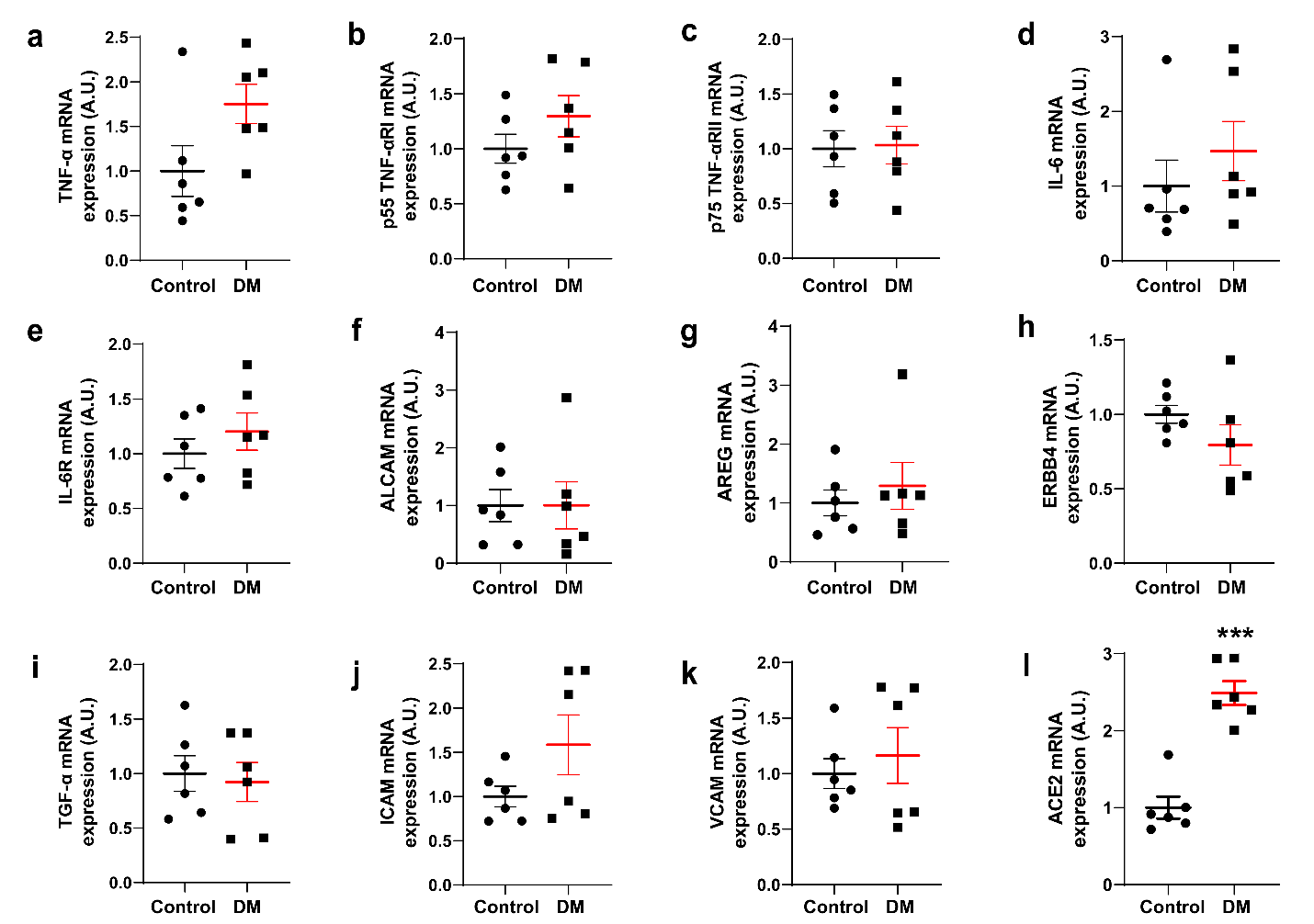


**Supplementary Figure S4.** **Comparison of mRNA expression of major ADAM17 substrates between diabetic and control mice. (a)** TNF-α mRNA expression. **(b)** P55 TNF-αRⅠmRNA expression. **(c)** P75 TNF-αRⅡmRNA expression. **(d)** IL-6 mRNA expression. **(e)** IL-6R mRNA expression. **(f)** ALCAM mRNA expression. **(g)** AREG mRNA expression. **(h)** ERBB4 mRNA expression. **(i)** TGF-α mRNA expression. **(j)** ICAM mRNA expression. **(k)** VCAM mRNA expression. **(l)** ACE2 mRNA expression. Data were mean± SEM. ****P* <0.001 vs. control group. n=6/group. DM: ADAM17^fl/fl^ diabetic mice, Control: ADAM17^fl/fl^ normal control mice.

**
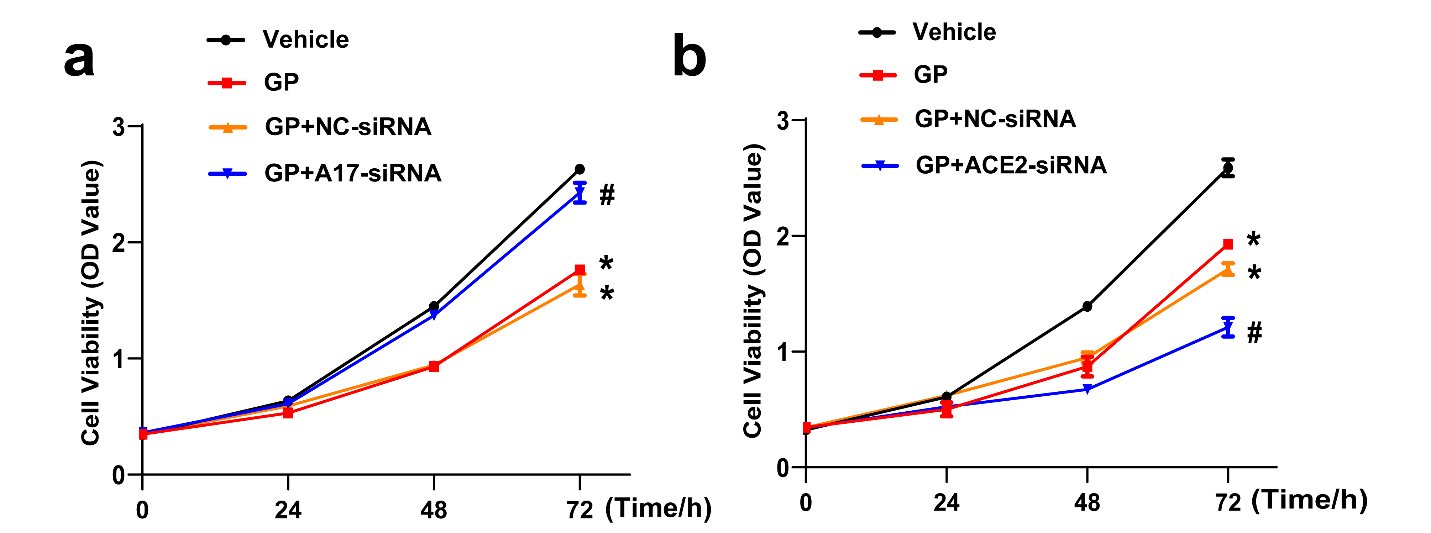
**

Supplementary Figure S5. Effects of ADAM17 and ACE2 knockdown on cell viability in differentiated H9c2 cells. (a) Cell viability in four groups of differentiated H9c2 cells treated with vehicle, GP, GP+NC-siRNA and GP+ADAM17-siRNA. (b) Cell viability in four groups of differentiated H9c2 cells treated with vehicle, GP, GP+NC-siRNA and GP+ACE2-siRNA. Five independent experiments were performed to derive the mean values. Data were mean± SEM. *P <0.05 vs. vehicle group. #P <0.05 vs. GP+NC-siRNA group.

**Supplementary Table 1. Body weight, blood glucose and serum lipid profile in mice**

|  | ADAM17^fl/fl^ Control (n=10) | A17^α-MHCKO^ Control (n=10) | ADAM17^fl/fl^ DM (n=10) | A17^α-MHCKO^ DM (n=10) |
| --- | --- | --- | --- | --- |
| BW (g) | 27.95±1.37 | 28.37±1.59 | 33.66±1.97* | 34.55±2.42* |
| FBG (mmol/L) | 7.45±1.18 | 8.01±1.52 | 28.13±6.61* | 29.33±2.06* |
| TC (mmol/L) | 3.28±0.86 | 3.49±0.78 | 4.52±0.51* | 4.01±1.026* |
| TG (mmol/L) | 1.55±0.28 | 1.57±0.31 | 2.07±0.32* | 1.79±0.36 |
| HDL-C (mmol/L) | 0.81±0.72 | 0.88±0.53 | 0.98±0.26 | 0.75±0.44 |
| LDL-C (mmol/L) | 2.17±0.32 | 2.15±0.46 | 2.20±0.51 | 2.49±0.42 |

Data were mean ± SD; **p* < 0.05 vs. ADAM17^fl/fl^ control group. BW: body weight; FBG: fasting blood glucose; TC: total cholesterol; TG: triglycerides; HDL-C: high-density lipoprotein cholesterol; LDL-C: low-density lipoprotein cholesterol; ADAM17^fl/fl^ control: ADAM17^fl/fl^ non-diabetic mouse group; A17^α-MHCKO^ control: A17^α-MHCKO^ non-diabetic mouse group; ADAM17^fl/fl^ DM: ADAM17^fl/fl^ diabetic mouse group; A17^α-MHCKO^ DM: A17^α-MHCKO^ diabetic mouse group.

**Supplementary Table 2. Genotyping primers for ADAM17^fl/fl^ and α-MHC-Cre mice.**

| Genotype | Sequence 5’-3’ | Primers |
| --- | --- | --- |
| ADAM17^fl/fl^ | TCC CCC AGG TAG ATT GTT TG | Forward |
|  | AGG ACC CAG GTT CAG TTC CT | Reverse |
| α-MHC-Cre | ATT TGC CTG CAT TAC CGG TC | Forward |
|  | ATC AAC GTT TTC TTT TCG G | Reverse |

α-MHC-Cre mice: alpha-myosin heavy chain-Cre mice.

**Supplementary Table 3. Primers used for RT-PCR analysis**.

| Gene | Sequence 5’-3’ | Primers |
| --- | --- | --- |
| ADAM17 (Mus) | AGGACGTAATTGAGCGATTTTGG | Forward |
|  | TGTTATCTGCCAGAAACTTCCC | Reverse |
| TNF-α (Mus) | AAGCAAGCAGCCAACCAG | Forward |
|  | TCTTCTGCCAGTTCCACG | Reverse |
| IL-6 (Mus) | AGTTGCCTTCTTGGGACTGA | Forward |
|  | TCCACGATTTCCCAGAGAAC | Reverse |
| ACE2 (Mus) | CAACCCAAAGAACCCACAAG | Forward |
|  | CTCTTCATACAACGGCCTCAG | Reverse |
| ICAM (Mus) | GTGATGCTCAGGTATCCATCCA | Forward |
|  | CACAGTTCTCAAAGCACAGCG | Reverse |
| VCAM (Mus) | GCAAAGGACACTGGAAAAGAG | Forward |
|  | TCAAAGGGATACACATTAGGGAC | Reverse |
| P55 TNF-α RI (Mus) | GCTGTTGCCCCTGGTTATCT | Forward |
|  | ATGGAGTAGACTTCGGGCCT | Reverse |
| P75 TNF-α RII (Mus) | GGTCAAGTGAGGGCTGAGAC | Forward |
|  | CCTAACCCGGACATGCACTT | Reverse |
| IL-6R (Mus) | TACGCTAGTGACACTTTCTCACA | Forward |
|  | TTCCGCTTTTTGCCTGAAGTC | Reverse |
| TGF-alpha (Mus) | CACTCTGGGTACGTGGGTG | Forward |
|  | CACAGGTGATAATGAGGACAGC | Reverse |
| AREG (Mus) | TTGCTGCTGGTCTTAGGCTC | Forward |
|  | TGGTCCCCAGAAAGCGATTC | Reverse |
| Alcam (Mus) | TGTCTCTGCGAATGAAAACAGA | Forward |
|  | GTAGACGACACCAGCAACGA | Reverse |
| Erbb4 (Mus) | ACGGGCCATTCCACTTTACC | Forward |
|  | CTGCCAGATCCCGATGAACA | Reverse |
| β-actin (Mus) | CACTGTGCCCATCTACGA | Forward |
|  | GTAGTCTGTCAGGTCCCG | Reverse |
| TFEB (Mus) | AAGGTTCGGGAGTATCTGTCTG | Forward |
|  | GGGTTGGAGCTGATATGTAGCA | Reverse |
| β-actin (Rattus) | CCACACCCGCCACCAGTTCG | Forward |
|  | TACAGCCCGGGGAGCATCGT | Reverse |
| ADAM17 (Rattus) | GGCCCTTTGAAGAGGTGAGG | Forward |
|  | CATGTGAAGGCCAAAACCCC | Reverse |
| ACE2 (Rattus) | TCCACTGAAGCTGGGCAGAA | Forward |
|  | AGCCAGACAAACAATGGTTGGAA | Reverse |

**Supplementary Table 4: Key resources of experiments *in vivo* and *in vitro***

|  | | |
| --- | --- | --- |
| **Antibodies** | **Source** | **Identifier** |
| **Western Blot** | | |
| ADAM17 (1:1000) | Abcam | Cat#ab2051; RRID: AB_302796 |
| ACE2 (1:1000) | Abcam | Cat#ab108252; RRID: AB_10864415 |
| LC3B (1:1000) | Abcam | Cat#ab48394; RRID: AB_881433 |
| Bcl2 (1:1000) | Abcam | Cat# ab32124; RRID: AB_725644 |
| Bax (1:1000) | Abcam | Cat# ab32503; RRID: AB_725631 |
| β-actin (1:1000) | Abcam | Cat# ab8227; RRID: AB_2305186 |
| AMPK (1:1000) | Cell Signaling Technology | Cat#5831; RRID: AB_10622186 |
| phosphorylated AMPK (1:1000) | Cell Signaling Technology | Cat#2535; RRID: AB_331250 |
| SQSTM1/p62 (1:1000) | Abcam | Cat# ab109012; RRID: AB_2810880 |
| cleaved caspase 3 (1:1000) | Cell Signaling Technology | Cat#9664; RRID: AB_2070042 |
| transcription factor EB (TFEB) (1:1000) | Proteintech | Cat#13372-1-AP; RRID: AB_2199611 |
| nuclear matrix protein p84 (1:1000) | GeneTex | Cat# GTX70220; RRID: AB_372637 |
| Atg3 (1:1000) | Cell Signaling Technology | Cat#3415; RRID: AB_2059244 |
| Atg5 (1:1000) | Cell Signaling Technology | Cat#12994; RRID: AB_2630393 |
| Atg7 (1:1000) | Cell Signaling Technology | Cat#8558; RRID: AB_10831194 |
| Atg12 (1:1000) | Cell Signaling Technology | Cat#4180; RRID: AB_1903898 |
| 4EBP1 (1:1000) | Cell Signaling Technology | Cat#9644; RRID: AB_2097841 |
| p-4EBP1 (1:1000) | Cell Signaling Technology | Cat#2855; RRID: AB_560835 |
| p70S6K (1:1000) | Cell Signaling Technology | Cat#9202; RRID: AB_331676 |
| p-p70S6K (1:1000) | Cell Signaling Technology | Cat#9204; RRID: AB_2265913 |
| mTOR (1:1000) | Cell Signaling Technology | Cat#2983; RRID: AB_2105622 |
| p-mTOR (1:1000) | Cell Signaling Technology | Cat#5536; RRID: AB_10691552 |
| ADAM17 (1:1000) | Bioss | Cat#bsm-52009R; |
| beclin-1 (1:1000) | Cell Signaling Technology | Cat#3495; RRID: AB_1903911 |
| cardiac troponin I (1:1000) | Abcam | Cat# ab47003; RRID: AB_869982 |
| myosin heavy chain 6 (MYH6) (1:1000) | Abcam | Cat# ab185967; RRID: AB_2890009 |
| HIF-1α | Proteintech | Cat#20960-1-AP; RRID: AB_10732601 |
| ADRA1A | Proteintech | Cat#19777-1-AP; RRID: AB_10643238 |
| **Immunofluorescence** | | |
| cTnT (1:100) | Abcam | Cat#ab8295; RRID: AB_306445 |
| PCM-1 (1:100) | Cell Signaling Technology | Cat#5213; RRID: AB_10556960 |
| Vimentin (1:100) | Cell Signaling Technology | Cat#5741; RRID: AB_10695459 |
| TFEB (1:100) | Proteintech | Cat#13372-1-AP; RRID: AB_2199611 |
| **Other reagents** | | |
| Product Name | Source | Identifier |
| DAPI | Abcam | Cat# ab104139 |
| TMRM (100nM) | Abcam | Cat# ab228569 |
| Hoechst 33342 (1μg/ml) | KeyGEN | Cat# KGA212 |
| Palmitic acid | Sigma | Cat# 292125 |
| BCA protein assay kit | Cwbio | Cat# CW0014 |
| chloroquine (CQ) | MCE | Cat# HY-17589A |
| ADAM17 siRNA (5’-3’: CCUGCGACUUGAGAAGCUUTT) | | |
| ACE2 siRNA (5’-3’: CCATCAAGCGTCAACTGAA) | | |
| HIF-1α siRNA (5’-3’: CTGATAACGTGAACAAATA) | | |
